# Achilles tendinopathy treatment via circadian rhythm regulation

**DOI:** 10.1016/j.jare.2024.10.022

**Published:** 2024-10-21

**Authors:** Yibo Zhang, Yizhang Wu, Yong Wang, Jun Lu, Yang Lu, Peng Wang, Lan Li, Wenjin Yan, Hongling Cai, Weisbecker Hannah Leigh, Lin Zhang, Wubin Bai, Qing Jiang, Xingquan Xu

**Affiliations:** a State Key Laboratory of Pharmaceutical Biotechnology, Division of Sports Medicine and Adult Reconstructive Surgery, Department of Orthopedic Surgery, Nanjing Drum Tower Hospital, Affiliated Hospital of Medical School, Nanjing University, Nanjing 210008, China; b Department of Applied Physical Sciences, University of North Carolina at Chapel Hill, Chapel Hill, NC 27599, USA; c Wide Bandgap Semiconductor Technology Disciplines State Key Laboratory, School of Microelectronics, Academy of Advanced Interdisciplinary Research, Xidian University, Xi’an 710071, China; d Jiangsu Key Laboratory for Biomaterials and Devices, State Key of Laboratory Bioeletronics, School of Biological Sciences & Medical Engineering, Southeast University, Nanjing 210096, China; e Jiangsu Engineering Research Center for 3D Bioprinting, Nanjing 210000, China; f National Laboratory of Solid State Microstructures, Collaborative Innovation Center of Advanced Microstructures, School of Physics, Nanjing University, Nanjing 210093, China; g Institute of Medical 3D Printing, Nanjing University, Nanjing 210000, China

**Keywords:** Achilles tendinopathy, Circadian clock, Bmal1-Nrf2 axis, Schottky heterojunction, Time of administration

## Abstract

**Introduction:**

Achilles tendinopathy (AT) is a prevalent musculoskeletal disorder closely linked to oxidative stress. Existing evidence suggests a potential link between circadian clock rhythms and oxidative stress. However, the precise role of the circadian clock in the progression and treatment of AT remains unclear.

**Objective:**

The purpose of this study was to investigate the role of the Achilles tendon circadian clock in AT pathology and explore the potential use of biomaterials for modulating the circadian clock in the treatment of AT.

**Methods:**

We utilized in vivo and in vitro models to investigate the alterations of the circadian clock within the Achilles tendon during the progression of AT, as well as its impact on disease development. Additionally, we fabricated Nb_2_C@CeO_2_ composites featuring a Schottky heterojunction for regulating the circadian rhythm and validated its therapeutic efficacy and molecular mechanism of AT through both in vivo and in vitro experiments.

**Results:**

The Achilles tendon functioned as a peripheral oscillator with an independent and self-sustained time-keeping system. The rhythm of the Achilles tendon clock was disrupted during the development of AT, as indicated by the decreased amplitude of *Bmal1* and *Nrf2* rhythm expression. Mechanistically, the knockdown of *Bmal1* disrupted the Achilles tendon clock, thereby destroying the *Bmal1*-*Nrf2* axis dependent molecular defense mechanism, and exacerbating the inflammatory response, whereas overexpression of *Bmal1* had a protective effect. Nb_2_C@CeO_2_ composites with Schottky heterojunctions enhance intercellular electrical signaling, boosting *Bmal1* expression and mitigating AT’s pathological changes. Importantly, enhancing *Bmal1* expression during its peak, rather than its trough, was more effective.

**Conclusion:**

This study identified the protective role of the circadian clock against oxidative stress and inflammation in the Achilles tendon. Achilles tendon circadian clock-targeted therapy represents a promising strategy for AT treatment.

## Highlights

• AT development disrupts Achilles tendon clock, reducing the amplitude of *Bmal1* and *Nrf2* rhythm expression.
• Disruption of *Bmal1-Nrf2* axis exacerbates AT inflammation; Boosting *Bmal1* mitigates tendinopathy.
• Schottky heterojunction boost *Bmal1* expression by amplifying intercellular electrical signals, thereby mitigating AT pathology.
• Timing-adjusted administration optimizes AT relief.

## Graphical abstract

**Figure:**
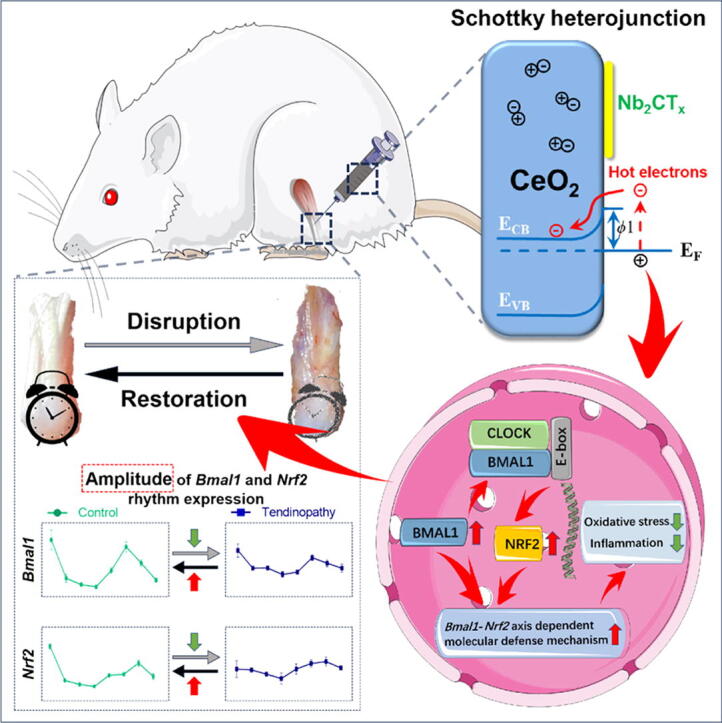

## Introduction

Achilles tendinopathy (AT) is a prevalent motor system disease that significantly reduces the quality of life for patients [1]. The current clinical treatments rely on physical therapy, surgical intervention, non-steroidal anti-inflammatory drugs, and corticosteroid injections; however, these interventions only offer partial relief of symptoms and may result in adverse side effects [2], [3], [4]. Consequently, there is an urgent demand to develop novel molecular targets and tailored drugs or biomaterials to advance therapeutic strategies for AT. Oxidative stress and inflammatory cytokines are believed to participate in AT pathogenesis [5]. The recent studies have provided evidence for the crucial role of the cellular molecular clock in governing oxidative stress and inflammatory cytokines across diverse tissue types [6], [7], [8]. However, whether this mechanism exists in Achilles tendon tissue remains unstudied.

Mammals rely on intricate circadian clock networks to synchronize daily metabolic and physiological rhythms, resulting in cyclic peaks and troughs known as circadian rhythms occurring within a 24-hour period [9], [10], [11]. The production of a circadian rhythms relies on the synchronized expression of a set of clock genes, primarily including the transcriptional activators aryl hydrocarbon receptor nuclear translocator-like (*Arntl*/*Bmal1*), circadian locomotor output cycles kaput (*Clock*), period 1/2/3 (*Per1/2/3*), etc [12], forming a transcription-translation feedback loop (TTFLs) [13]. BMAL1, the core regulator of the molecular clock, forms a heterodimeric partnership with CLOCK and binds to the E-box sites throughout the genome, thereby inducing rhythmic expression of circadian clock control genes [14], [15], [16]. Previous studies have demonstrated that *Bmal1* can regulate the expression of Nuclear factor erythroid 2-related factor 2 (*Nrf2*) in various tissues [17]. NRF2, a transcription factor belonging to the basic leucine zipper (bZIP) family, plays a crucial role in regulating antioxidant protein expression [18], [19], [20]. Therefore, aberrant *Bmal1* expression disrupts the antioxidant defense mechanism mediated by the *Bmal1*-*Nrf2* axis and leads to various diseases [21], [22]. *Nrf2* is expressed in Achilles tendon cells and has been shown to suppress the production of inflammatory factors by inhibiting reactive oxygen species (ROS) [23]. However, the involvement of the circadian rhythm-based *Bmal1*-*Nrf2* axis-mediated antioxidant responses molecular mechanisms in Achilles tendon remains largely unexplored. Therefore, the urgent need lies in comprehending the mechanisms underlying circadian clock disruption in AT development and formulating therapeutic strategies for regulating the circadian clock of Achilles tendon.

Currently, the regulation of the circadian clock has been implemented in the treatment of various diseases by employing drugs that enhance *Bmal1* expression, such as melatonin (Mel) [24]. However, therapeutic strategies employing pharmacological modulation of *Bmal1* expression are constrained by the limited drug half-life and their susceptibility to interference from the microenvironment [25]. Therefore, it is imperative to develop biomaterials capable of efficiently and durably regulating *Bmal1* expression in order to precisely control the cellular circadian clock and facilitate restoration of cellular physiological functions. Given that BMAL1 is a Per-Arnt-Sim (PAS) domain protein, the PAS domain possesses the capability to perceive oxygen, REDOX potential, and light and is expressed in various proteins involved in electrical signal sensing [26], [27]. Based on this, we proposed the hypothesis that *Bmal1* expression might be regulated by alterations in intercellular electrical signals. The carbon-based two-dimensional (2D) MXene nanomaterials, niobium carbide (Nb_2_C), and the semiconductor CeO_2_ NPs have gained significant attention in biomedical applications due to their exceptional biocompatibility, versatility, and stability [28], [29], [30]. To enhance the expression level of the *Bmal1*, we constructed a Schottky heterojunction model Nb_2_C@CeO_2_ with a Schottky interface utilizing the aforementioned two materials. The Schottky interface modulates and/or amplifies the electrical signal by the directional transfer of endogenous electrons around the Schottky interface [31], thereby augmenting *Bmal1* expression and enhancing the antioxidant response mediated by the *Bmal1*-*Nrf2* axis for AT treatment.

In this study, we sought to investigate the role of circadian rhythms in the treatment of AT. Initially, we discovered that tendons act as peripheral oscillators and exhibit rhythmic disturbances in tissues and cells affected by tendinopathy. Regulating circadian rhythms resulted in effective therapeutic effects for AT. Additionally, we developed an injectable biomaterial based on Nb_2_C@CeO_2_ with Schottky heterojunctions capable of regulating the expression of *Bmal1* to modulate circadian rhythms and verified their effectiveness in treating tendinopathy. Our in vivo studies further elucidate the impact of varying administration times on the therapeutic outcomes of AT. This has practical clinical significance and provides guidance for the current clinical scenario where the optimal administration times for numerous conditions, including AT, remain undefined. In summary, our research offers novel insights into the pathogenesis of AT and proposes potential therapeutic strategies, which can serve as valuable practical references for future interventions.

## Material and methods

### Experimental design

The purpose of this study was to investigate the role of the Achilles tendon circadian clock in AT pathology and explore the potential use of biomaterials for modulating the circadian clock in the treatment of AT. We examined the function, mechanisms, and clinical significance of the Achilles tendon circadian clock to address the following questions: (i) Is the Achilles tendon a peripheral oscillator? (ii) What is the role and mechanism of circadian rhythm disruption in the progression of AT? (iii) Can Schottky heterojunction be used to regulate the circadian rhythm for treating AT? (iv) Can better therapeutic outcomes be achieved by adjusting the timing of drug administration? In this study, we collected human Achilles tendon samples and established an Achilles tendon disease model in Sprague Dawley (SD) rats and tenogenic differentiation of stem cells (TDSCs). We evaluated the impact of Achilles tendon disease on the Achilles tendon clock. We modulated the Achilles tendon clock at both the animal and cellular levels using light deprivation, siRNA transfection, and plasmid overexpression techniques to observe the effects of the Achilles tendon clock on AT. Furthermore, we investigated the feasibility of treating AT by enhancing *Bmal1* expression using newly developed materials with interface Schottky barrier. Finally, we identified optimal timing for drug administration to achieve improved therapeutic effects. Detailed materials and methods are described in Supporting Information.

### Human studies

Tendon tissues (n = 6) were obtained from patients who underwent arthroscopic surgery and total knee replacement surgery with motor system injury including tendinopathy (n = 3) and normal tendon tissues obtained from amputated patients (n = 3) at Nanjing Drum Hospital. The clinical information of these patients is listed in Table S1. The study protocol received approval from the Ethics Committee of Nanjing Drum Hospital. Acquisition of human materials followed informed consent procedures, and all research activities adhered to the principles delineated in the Declaration of Helsinki.

### Cell studies

The TDSCs were isolated and cultured as previously described [32]. Tert-Butyl Hydroperoxide (TBHP) was used to induce inflammation in TDSCs as previously described [32]. Following the establishment of the model or subsequent treatment, cells were harvested for assessment via Cell Counting Kit-8 (CCK-8) assay, live/dead cell staining, reactive oxygen species (ROS) detection, quantitative polymerase chain reaction (qPCR), Western blot analysis, and immunofluorescence staining. To study the circadian clock system of TDSCs, the cells were treated with 50 % horse serum (HyClone, Logan, UT, USA) for 2 h [33]. The medium was subsequently replaced with DMEM medium supplemented with 1 % penicillin–streptomycin solution and 10 % fetal bovine serum. Subsequently, the protein and mRNA were extracted every 4 h, 8–36 h after serum shock, and detected by qPCR and Western blot analysis. For knockdown or overexpression of *Bmal1*, TDSCs were transfected with siRNA duplexes targeting *Bmal1* or overexpressing *Bmal1* plasmids.

### Animal studies

The Sprague Dawley (SD) male rats (8 weeks old) were housed in a controlled environment with stable temperature and humidity under a 12/12 h light/dark cycle (LD). To investigate whether the Achilles tendon is a peripheral oscillator, rats were euthanized by cervical dislocation, and tendons were collected at specific time points (ZT1, ZT5, ZT9, ZT13, ZT17 and ZT21) for qPCR and **WB**. To study the pathological changes and circadian rhythm changes in AT, a solution of collagenase I was injected into the left Achilles bone-tendon junction every 2 days at Zeitgeber Time 1 (ZT1) to induce tendon injury. After 14 days, at the time point of ZT1, tendons were extracted and prepared for qPCR, Western blot analysis, HE, Masson, immunofluorescence, and immunohistochemical staining. To investigate the effect of circadian dysrhythmia on AT, we employed light deprivation as a model of circadian dysrhythmia[34] and induced AT in rats under normal light (LD) and constant darkness (DD) conditions. To validate of the efficacy of *Bmal1* enhancer Schottky heterojunction, rats were randomly divided into four groups:(i) Control group; (ii)Tendinopathy group; (iii) Nb_2_C group and (iv) Schottky group. Except for the control group, all rats in the other groups were injected with 100 μL of a 5 mg/mL solution of type I collagenase at the tendon-bone junction of the left Achilles tendon every two days to induce tendon injury. After 14 days, 50 μL of Nb_2_C (80 mg/mL) (Nb_2_C group), Nb_2_C@CeO_2_ (80 mg/mL) (Schottky group), or PBS (Control group and Tendinopathy group) was injected into the left Achilles tendon respectively, at Zeitgeber time (ZT) 1 daily. After another 14 days, Achilles tendon tissue samples were collected from all groups at ZT1 for subsequent experiments. To investigation of the effect of different administration times on efficacy, rats were divided into four groups: (i) Control group; (ii)Tendinopathy group; (iii) Schottky (ZT13) group; (iv) Schottky (ZT1) group. AT was induced using established methods, and 50 μl of Nb_2_C@CeO_2_ (80 mg/ml) were injected at the left Achilles bone-tendon junction at the corresponding time points (ZT13/ZT1) daily. After 14 days, SD male rats were euthanized by cervical dislocation, and Achilles tendon tissue samples were collected from all groups at ZT1 for subsequent experiments. All animal experiments were conducted strictly in accordance with the Guide for the Care and Use of Laboratory Animals and obtained approval from the Animal Investigation Ethics Committee of Nanjing Drum Tower Hospital.

## Results

### The Achilles tendon functions as a peripheral oscillator

**Fig. 1:**
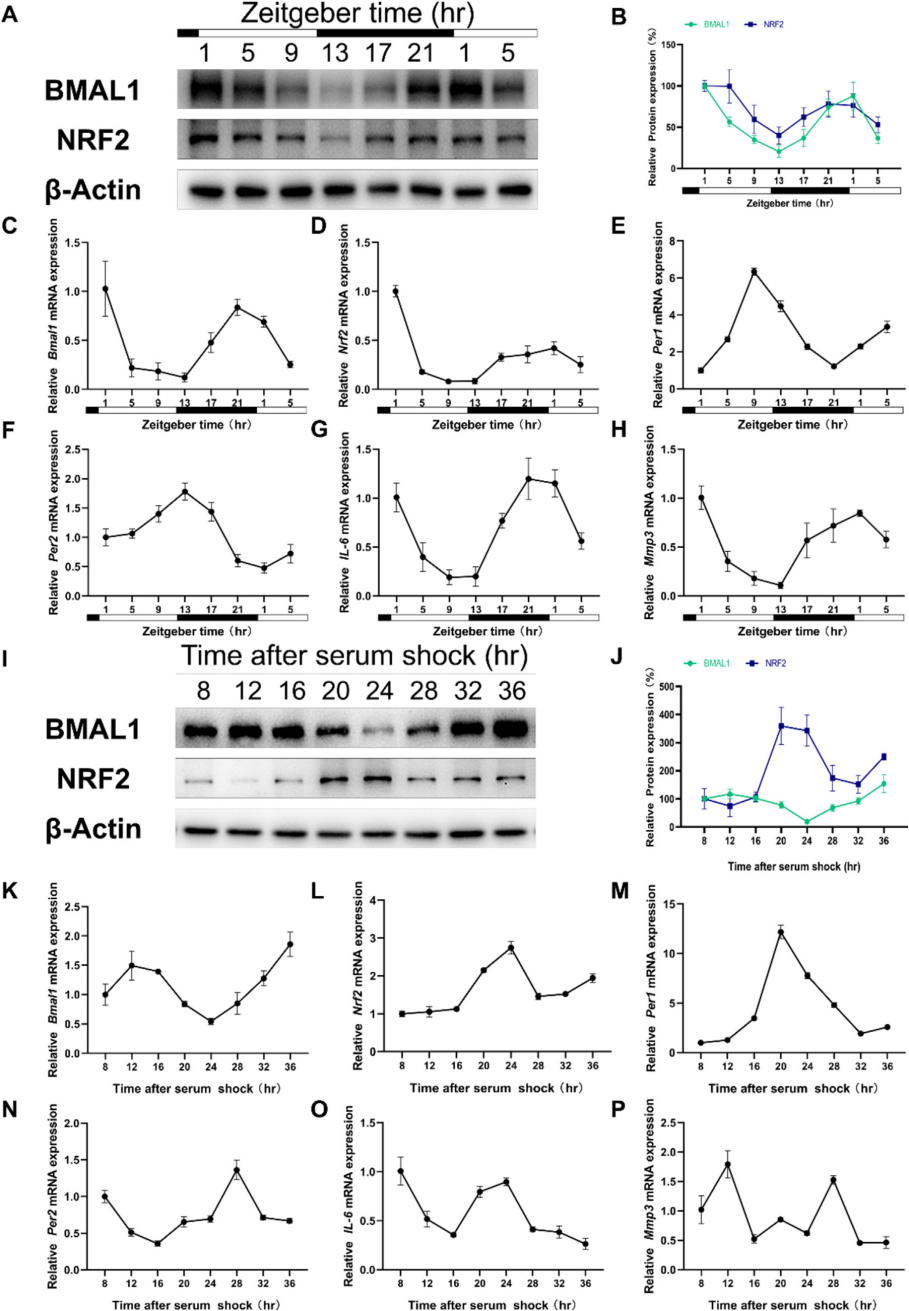
The Achilles tendon functions as a peripheral oscillator. (A and B) Western blot analysis of BMAL1 and NRF2 at 4-hour intervals over a 28-hour period in Achilles tendon tissues. At each time point, three to five rats were euthanized for sampling. All the rats were bred on a WT SD background. (C to H) The mRNA expression of *Bmal1*, *Nrf2*, *Per1*, *Per2*, *IL-6,* and *Mmp3* in Achilles tendon tissues is shown. (I and J) Western blot analysis of BMAL1 and NRF2 at 4-hour intervals over a 28-hour period in TDSCs. (K to P) The mRNA expression of *Bmal1*, *Nrf2*, *Per1*, *Per2*, *IL-6* and *Mmp3* in TDSCs is shown. Data are presented as the mean ± SD.

We first examined the potential impact of time of day on the protein expression of BMAL1 and NRF2 in the Achilles tendon of a rat model organism. As expected, we observed a rhythmic pattern in clock gene expression for BMAL1 and a significant diurnal rhythm in NRF2 expression (Fig. 1**A,B**). Specifically, BMAL1 and NRF2 proteins peaked at “zeitgeber” time 1 (ZT1) and reached a trough at ZT13, which were further confirmed by immunofluorescence staining (Figure S1A,B). Consistent with protein expression levels, *Bmal1* and *Nrf2* mRNA also peaked at ZT1 and troughed at ZT13 (Fig. 1C,D). Furthermore, other genes, including *Per1*, *Per2*, Interleukin-6 (*IL-6*), and Matrix metalloproteinase-3 (*Mmp3*) also exhibited robust rhythmic patterns (Fig. 1E-H). Interestingly, the rhythmic expression of *IL-6* and *Mmp3* in the Achilles tendon appeared to align with the rhythmic expression of *Bmal1* and *Nrf2*.

To eliminate the effects of light and/or cell-extrinsic factors, we conducted a serum shock protocol in tendon-derived stem cells (TDSCs) [33]. Clear rhythms in the expression of *Bmal1* (Figure S1C) and *Per2* (Figure S1D) were observed in TDSCs, particularly at 8–36 h after serum shock. Accordingly, we examined the protein expression levels of BMAL1 and NRF2 at 8–36 h after serum shock (Fig. 1I,J). In contrast to the in vivo Achilles tendon tissue, the protein levels in TDSC exhibit opposite rhythmic patterns, with BMAL1 peaking at 12 h after serum shock and NRF2 reaching a trough at the same time, and vice versa at 24 h (Fig. 1I,J and Figure S1E,F). The qPCR results exhibited consistent trends with the protein levels (Fig. 1K,L). Furthermore, the expression of *Per1* and *Per2* in TDSCs also demonstrated a strong rhythmic pattern (Fig. 1M,N). Strikingly, *IL-6* and Tumor Necrosis Factor-α (*Tnf-α*) mRNA expression rhythms resembled that of *Nrf2* (Fig. 1O and Figure S1G), while *Mmp3* and *IL-1β* mirrored *Bmal1* (Fig. 1P and Figure S1H). However, no significant rhythmic expression of Collagen type I (*Col1*) and Collagen type III (*Col3*) was observed in TDSCs (Figure S1I,J).

Given the rhythmic expression of circadian clock-related genes in the Achilles tendon both in vivo and in vitro, these findings suggest that the Achilles tendon may function as a peripheral oscillator. Considering the rhythmic expression of *Nrf2* in the Achilles tendon, it is plausible that the Achilles tendon clock may regulate *Nrf2*-mediated antioxidant and anti-inflammatory pathways in the Achilles tendon.

### Achilles tendinopathy is associated with a damped circadian rhythm

**Fig. 2:**
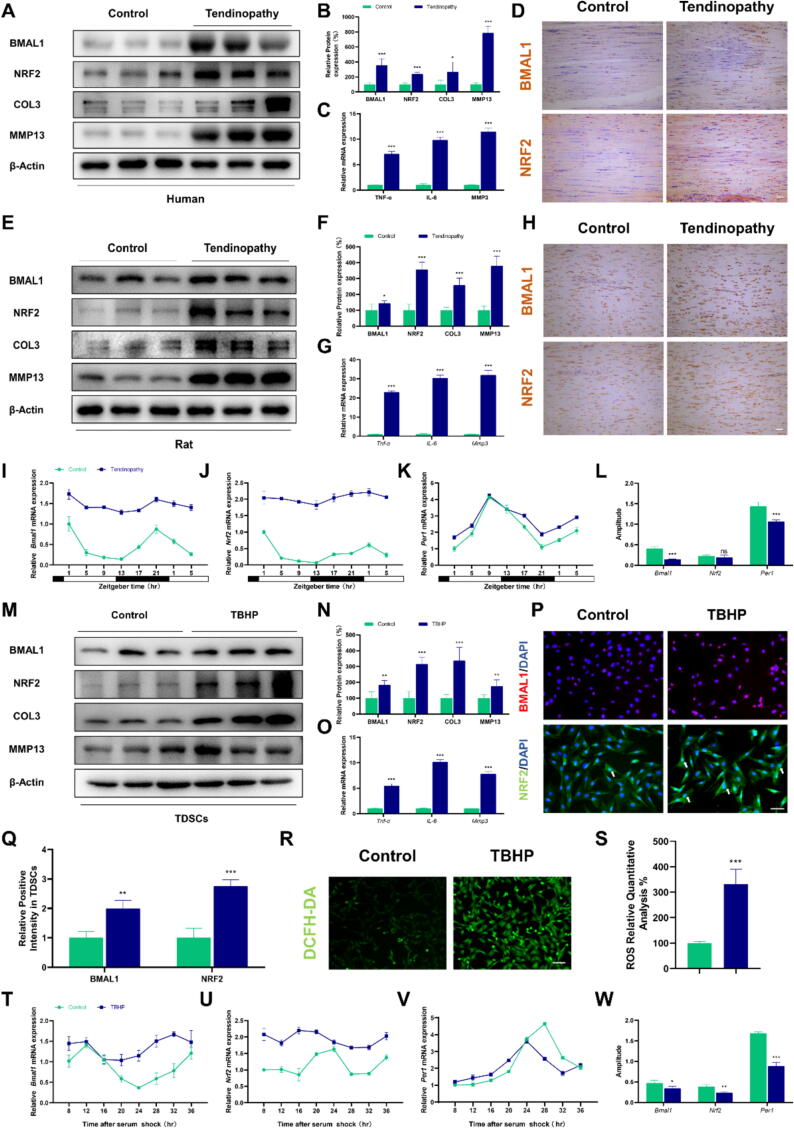
Unraveling a disrupted circadian clock in Achilles tendinopathy. (A and B) Western blot analysis of BMAL1 and NRF2 in human Achilles tendon tissues with or without tendinopathy. (C) The mRNA expression of *Tnf-α*, *IL-6,* and *Mmp3* in human Achilles tendon tissues with or without tendinopathy. (D) Representative images of immunochemistry analysis for BMAL1 and NRF2 in human Achilles tendon tissues with or without tendinopathy. (E and F) Western blot analysis of BMAL1 and NRF2 in rat Achilles tendon tissues with or without tendinopathy. (G) The mRNA expression of *Tnf-α*, *IL-6,* and *Mmp3* in rat Achilles tendon tissues with or without tendinopathy. (H) Representative images of immunochemistry analysis for BMAL1 and NRF2 in rat Achilles tendon tissues with or without tendinopathy. (I to K) The mRNA expression of *Bmal1*, *Nrf2,* and *Per1* at 4-hour intervals over a 28-hour period in rat Achilles tendon tissues with or without tendinopathy. (L) The amplitude of the mRNA rhythmic expression of *Bmal1*, *Nrf2* and *Per1* in rat Achilles tendon tissues with or without tendinopathy. (M and N) Western blot analysis of BMAL1 and NRF2 in TDSCs treated with or without TBHP. (O) The mRNA expression of *Tnf-α*, *IL-6,* and *Mmp3* in TDSCs treated with or without TBHP. (P and Q) The immunofluorescence staining analysis for BMAL1 and NRF2 in TDSCs treated with or without TBHP. The white arrow indicates NRF2 in the nucleus. (R and S) The ROS staining analysis in TDSCs treated with or without TBHP. (T to V) The mRNA expression of *Bmal1*, *Nrf2,* and *Per1* at 4-hour intervals over a 28-hour period in TDSCs treated with or without TBHP. (W) The amplitude of the mRNA rhythmic expression of *Bmal1*, *Nrf2,* and *Per1* in TDSCs treated with or without TBHP. Data are presented as the mean ± SD. *p < 0.05, **p < 0.01, ***p < 0.001; ns, not significant. Scale bar, 100 μm.

Normal and inflammatory tendon tissue from humans were collected for our study. The tendinopathy group showed significantly higher COL3 and MMP13 protein expression compared to the control group (Fig. 2**A,B**). qPCR analysis indicated elevated mRNA levels of *TNF-α*, *IL-6*, and *MMP3* in the tendinopathy group (Fig. 2C), indicating the presence of inflammation in tendinopathic tissues. Notably, BMAL1 and NRF2 protein expression was elevated in inflamed tendon tissues compared to healthy tendon tissues, as confirmed by immunohistochemical staining (Fig. 2D and Figure S2A). Similar trends were observed in the Type I collagenase-induced Achilles tendon disease model in SD rats (Fig. 2E-H and Figure S2B-D) [35]. More interestingly, the expression of BMAL1 and NRF2 gradually decreased during the late stages of Achilles tendinopathy development in rats (Figure S2E,F). Moreover, the oscillation of the mRNA expression of *Bmal1*, *Nrf2* and *Per1* displayed an amplitude-reducing phenotype in AT tissues compared with the healthy Achilles tendon tissues in rats (Fig. 2I-L), suggesting the disruption of rhythmic pattern [36]. Consistent with the in vivo results, TDSCs exhibited increased expression of *Bmal1* and *Nrf2* in the presence of inflammation (Fig. 2M-Q). Additionally, ROS staining results indicated excessive accumulation of ROS in TDSCs under inflammatory conditions (Fig. 2R-S). Importantly, TBHP induction also resulted in the amplitude reduction of rhythmic expression of *Bmal1*, *Nrf2* and *Per1* in TDSCs (Fig. 2T-W). Changes in the period were also studied (Figure S2G,H). These data collectively reveal that AT alters the clock residing in the Achilles tendon.

### Circadian misalignment exacerbates Achilles tendinopathy in rat

**Fig. 3:**
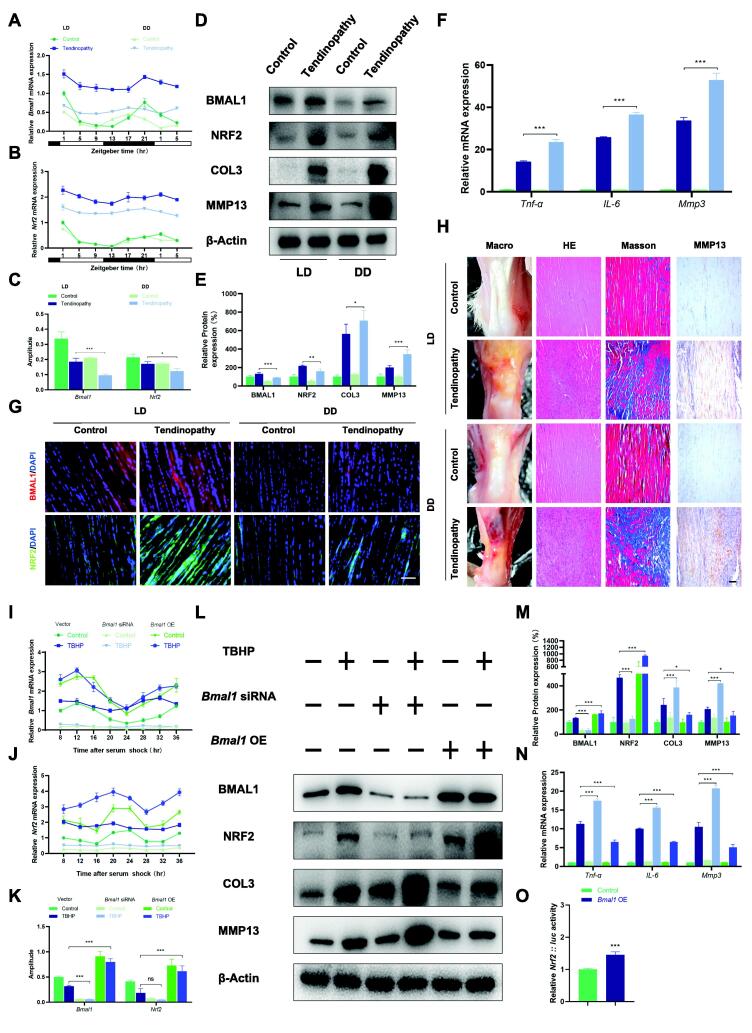
*Bmal1* is a potential therapeutic target for Achilles tendinopathy. (A and B) The mRNA expression of *Bmal1* and *Nrf2* at 4-hour intervals over a 28-hour period in rat Achilles tendon tissues with or without tendinopathy under LD or DD conditions. (C) The amplitude of the mRNA rhythmic expression of *Bmal1* and *Nrf2* in the four groups. (D and E) Western blot analysis of BMAL1, NRF2, COL3, and MMP13 in the four groups. (F) The mRNA expression of *Tnf-α*, *IL-6,* and *Mmp3* in rat Achilles tendon tissues. (G) The immunofluorescence staining analysis for BMAL1 and NRF2 in the four groups. (H) The Macro-picture, HE staining, Masson staining, and immunohistochemistry of MMP13 in the four groups (I and J) The mRNA expression of *Bmal1* and *Nrf2* at 4-hour intervals over a 28-hour period in TDSCs treated with *Bmal1* siRNAs or *Bmal1* overexpression plasmid with or without TBHP. (K) The amplitude of the mRNA rhythmic expression of *Bmal1* and *Nrf2* in the six groups. (L and M) Western blot analysis of BMAL1, NRF2, COL3, and MMP13 in the six groups. (N) The mRNA expression of *Tnf-α*, *IL-6,* and *Mmp3* in the six groups. (O) The luciferase analysis of *Nrf2* in TDSCs treated with or without *Bmal1* overexpression plasmid. Data are presented as the mean ± SD. *p < 0.05, **p < 0.01, ***p < 0.001; ns, not significant. Scale bar, 100 μm.

To investigate the effect of circadian dysrhythmia on AT, we employed light deprivation as a model of circadian dysrhythmia[34] and induced AT in rats under LD and constant darkness DD conditions. The qPCR results showed that constant darkness disrupted the Achilles tendon clock, reduced the amplitude of rhythmic mRNA expression of *Bmal1* and *Nrf2* in the Achilles tendon, and exacerbated the amplitude reduction caused by AT (Fig. 3**A-C** and Figure S3A). Western blot analysis and tissue immunofluorescence results revealed that circadian dysrhythmia caused a decrease in the protein expression of BMAL1 and NRF2 (Fig. 3D-G and Figure S3B). Notably, continuous darkness intensified the severity of AT, as indicated by increased protein expression of COL3 and Matrix metalloproteinase-13 (MMP13) (Fig. 3D,E) and elevated mRNA expression of *Tnf-α*, *IL-6*, and *Mmp3* (Fig. 3F). Histological analysis revealed that rats exposed to constant darkness exhibited aggravated symptoms of Achilles tendonitis (Fig. 3H). Furthermore, immunohistochemical staining demonstrated an increased proportion of MMP13-positive cells in the Achilles tendon tissue of rats with AT and these effects were further exacerbated by constant darkness (Fig. 3H and Figure S3C). Collectively, these findings suggest that circadian dysregulation induced by light deprivation reduces the expression and amplitude of *Bmal1* and *Nrf2* in the Achilles tendon, rendering rats more susceptible to AT.

### *Bmal1* plays a protective role in Achilles tendinopathy

To further elucidate the role of *Bmal1* in the occurrence and development of AT, we used small interfering RNAs (siRNAs) to knock down *Bmal1* in TDSCs, while utilizing pcDNA3.1(+)-Arntl-3xFLAG vectors to increase the expression of *Bmal1*. The results of qPCR showed that specific knockdown of *Bmal1* led to a reduction in the rhythmic expression amplitudes of *Bmal1* and *Nrf2*, whereas overexpression of *Bmal1* resulted in an increase in amplitudes (Fig. 3I-K and Figure S3D). Western blot analysis showed the same trend in the expression levels of BMAL1 and NRF2 proteins (Fig. 3L,M). Moreover, knocking down *Bmal1* led to more severe cellular damage, including increased mRNA expression of *Tnf-α*, *IL-6*, and *Mmp3* (Fig. 3N), elevated expression of COL3 and MMP13 proteins (Fig. 3L,M), as well as increased accumulation of ROS (Figure S3E,F). Conversely, overexpression of *Bmal1* attenuated the extent of these damages (Fig. 3L-N). These findings indicate that *Bmal1* plays a significant role in the antioxidant stress and anti-inflammatory processes in Achilles tendon tissue. Furthermore, luciferase assays revealed that overexpression of *Bmal1* in TDSCs significantly stimulated *Nrf2*:: luc activity (Fig. 3O), suggesting that *Bmal1* may exert its protective effect against oxidative stress by activating *Nrf2* transcription. As a result, promoting the expression of *Bmal1* may be a potential new therapeutic strategy for treating AT.

### Synthesis and Characterization of *Bmal1* enhancer Schottky heterojunction

**Fig. 4:**
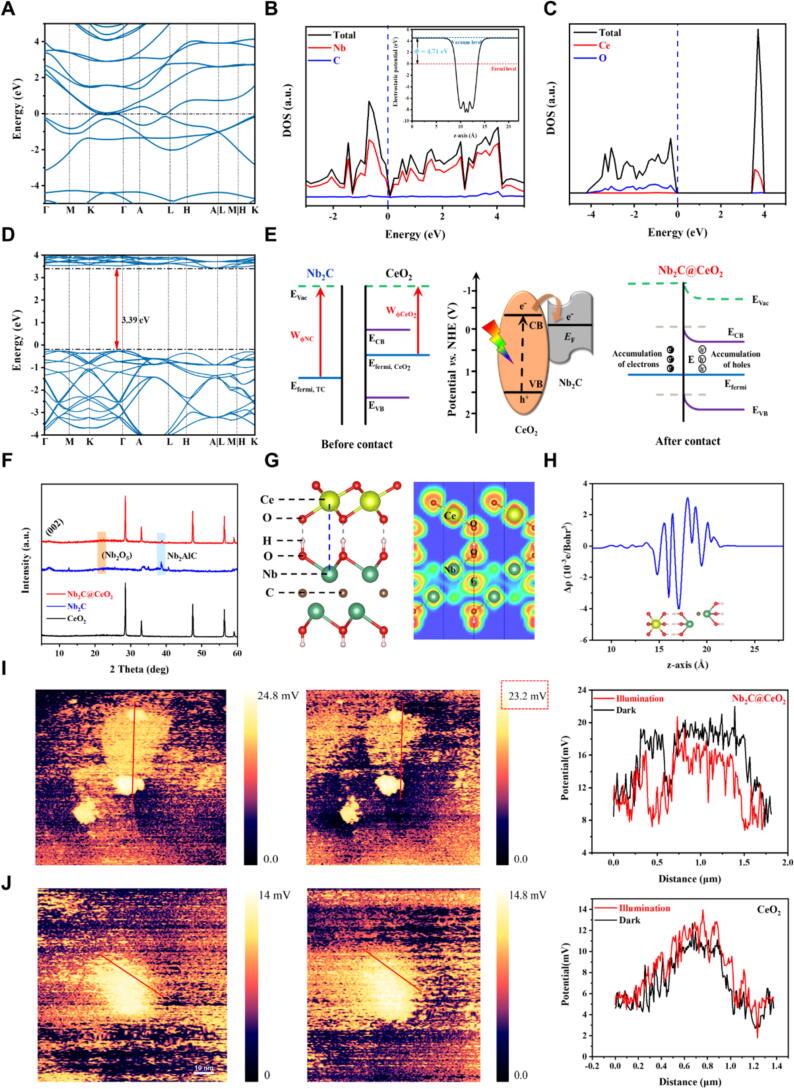
The construction of Schottky heterojunction and its corresponding regulation of electrical signals demonstrated by DFT calculation and photoelectric performance. (A) Band structure of Nb_2_C calculated by HSE06. (B) Density of states (DOS) plots for Nb_2_C. Inset: The planar average potential along the z-axis and work function of Nb_2_C. (C) Density of states (DOS) plots for CeO_2_. (D) Band structure and calculated excitation energy of CeO_2_. (E) Schematic illustration for charge carrier migration on Nb_2_C@CeO_2_ composite (Schottky heterojunction). (F) XRD patterns of CeO_2_, Nb_2_C, and Nb_2_C@CeO_2_ (Schottky heterojunction). (G) Stable configuration for Nb_2_C (OH)@ CeO_2_ and corresponding electron localization functions (ELFs). (H) Plane-averaged charge density difference Δρ along the z-direction. (I) KPFM potential images of Nb_2_C@CeO_2_ (Schottky heterojunction) in the dark and under illumination, corresponding to the measured surface potentials. (J) KPFM potential images of CeO_2_ in the dark and under illumination, corresponding to the measured surface potential. Data are presented as the mean ± SD. Scale bar, 10 nm.

We synthesized a Schottky heterojunction to conveniently and stably enhance *Bmal1* expression, leveraging its ability to amplify intercellular electrical signals. This Schottky heterojunction facilitated the creation of an interfacial Schottky built-in electric field, which impacts the circadian rhythm. Our Schottky construction was substantiated through density functional theory (DFT) calculations, revealing the band structure and excitation energy of Nb_2_C (Fig. 4**A,D**) and confirming its metallic nature with density of states (DOS) analysis (Fig. 4B,C). The Schottky barrier, as illustrated in Fig. 4E, was effectively formed due to appropriate positions of the conductive band (CB) and Fermi levels between Nb_2_C and CeO_2_. We successfully fabricated the Schottky heterojunction, and its morphology characteristics confirmed successful fabrication (Figure S4A-E). Additional analyses, including X-ray diffraction (XRD, Fig. 4F) and other internal structural measurements (XPS curves, Raman spectra, refer to Figure S5A-D) were conducted to investigate the internal structure and bonding condition of Schottky heterojunction. These results highlight the optimized electronic structure of the Schottky heterojunction, which is instrumental in modulating electrical signals efficiently.

Considering REDOX reactions in the humoral environment[37], we refined the Schottky model (Figure S5E-F) to utilize the most stable stacking configuration between Nb_2_C (OH) and CeO_2_, as shown in Fig. 4G. Electron localization functions (ELFs) were used to visualize the electron output in Schottky heterojunctions and suggested a weak interface due to the absence of a covalent bond between Nb_2_C (OH) and CeO_2_. The plane-averaged charge density difference (Fig. 4H) reveals charge accumulation near Nb_2_C (OH) and depletion near CeO_2_ at the interface, indicating charge transfer from CeO_2_ to Nb_2_C (OH). This phenomenon of directional electron transfer in the Schottky heterojunction alleviates carrier quenching and boosts the electrical signal intensity. The electrical signal strength on the Schottky heterojunction surface was measured using a Scanning Kelvin Probe Microscope (SKPM). Schottky heterojunction exhibited a surface potential of 24.8 mV in dark conditions (Fig. 4I) compared to pristine CeO_2_ NPs (Fig. 4J), indicating enhanced electron transfer. Also, it showed decreased surface photovoltage (SPV) under excitation, reflecting a higher concentration of electrons. The Schottky heterojunction enables the regulation of electrical signals through the Faradaic current and electrochemical reactions. Nb_2_C, due to its inherent ROS scavenging properties, can be used as a therapeutic modality for treating AT [28]. Therefore, Nb_2_C was utilized as a positive control to validate the therapeutic efficacy of Schottky heterojunction. CCK-8 assay (Figure S6A-C) and live/dead staining (Figure S6D) demonstrated the minimal cytotoxic effect of Nb_2_C and Schottky heterojunction on TDSCs. H&E staining in heart, liver, spleen, lung, kidney, and testis tissues (Figure S6E) and Elisa assay for interleukin-6 (IL-6) and C-reactive protein (CRP) levels in the serum (Figure S6F,G) confirmed the good biocompatibility of Nb_2_C and Schottky heterojunction in vivo.

### Schottky heterojunction increase *Bmal1* expression and activates *Nrf2*-driven antioxidant pathway

**Fig. 5:**
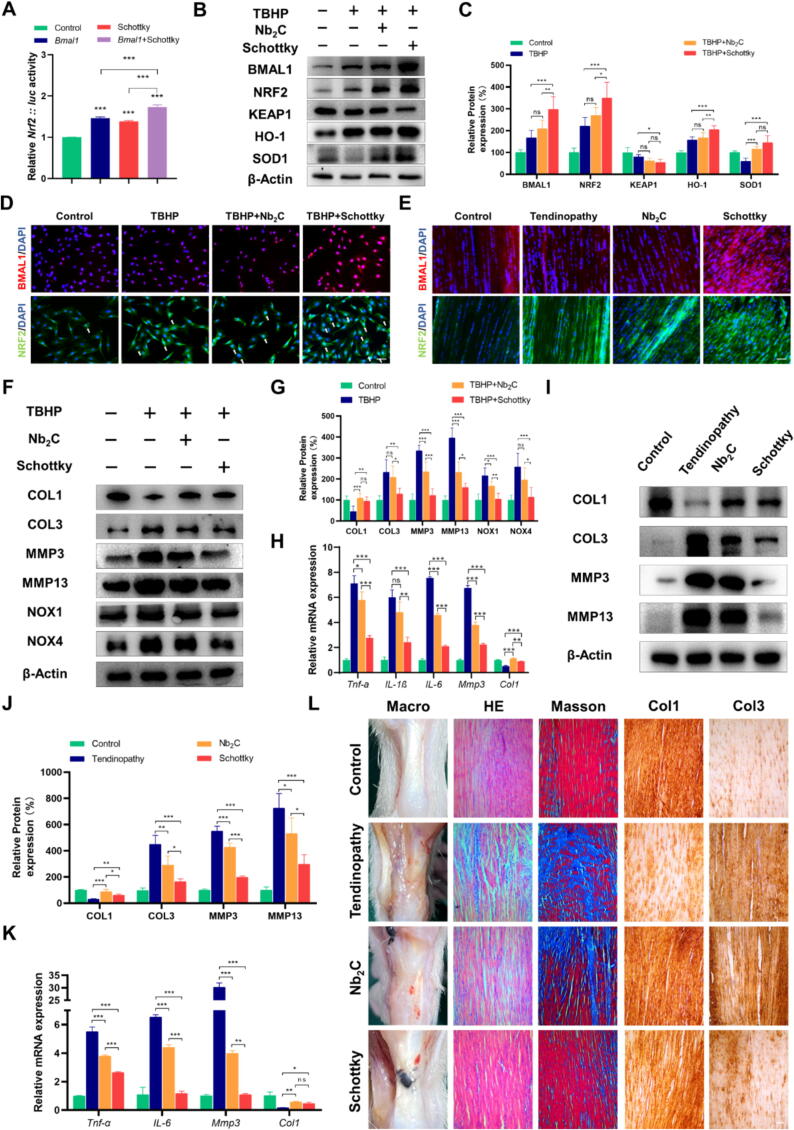
*Bmal1* enhancer Schottky heterojunction increases *Bmal1* expression and activates *Nrf2*-driven antioxidant pathway to ameliorates tendon deterioration in tendinopathy in vitro and in vivo. (A) The luciferase analysis of *Nrf2* in TDSCs treated with or without *Bmal1* overexpression plasmid or Schottky heterojunction. (B and C) Western blot analysis for BMAL1, NRF2, KEAP1, SOD-1, and HO-1 in TDSCs after TBHP treatment and treatment with or without Nb_2_C or Schottky heterojunction. (D) Immunofluorescence staining for BMAL1 and NRF2 in TDSCs in the four groups. The white arrow indicates NRF2 in the nucleus. (E) Immunofluorescence staining for BMAL1 and NRF2 in rat Achilles tendon tissues treated with or without Nb_2_C or Schottky heterojunction. (F and G) Western blot analysis for COL1, COL3, MMP3, MMP13, NOX1, and NOX4 in TDSCs after TBHP treatment and treatment with or without Nb_2_C or Schottky heterojunction. (H) Quantitative real-time PCR analysis of *Tnf-α*, *IL-1β*, *IL-6*, *Mmp3,* and COL1 mRNA levels in TDSCs in the four groups. (I and J) Western blot analysis for COL1, COL3, MMP3, and MMP13 in the tendon tissues of rats in the four groups (control, tendinopathy, Nb_2_C, and Schottky heterojunction). (K) Quantitative real-time PCR analysis of *Tnf-α*, *IL-6*, *Mmp3,* and COL1 mRNA levels in the four groups. (L) Macro-picture, HE staining of tendon, Masson staining, and immunohistochemistry staining of COL1a and COL3 in the tendon tissues of rats in the four groups. Data are presented as the mean ± SD. *p < 0.05, **p < 0.01, ***p < 0.001; ns, not significant. Scale bar, 100 μm.

To determine whether Schottky heterojunction could be used as a therapeutic approach to treat AT by enhancing *Bmal1* expression, we evaluated its effects on the stimulation of *Nrf2*: luc activity, and the luciferase assay results indicated that the overexpression of *Bmal1* in TDSCs led to a significant stimulation of *Nrf2*: luc activity, and this increase was further enhanced by Schottky heterojunction treatment (Fig. 5**A**). Then we evaluated its effects on the expression levels of BMAL1, NRF2, Kelch Like ECH Associated Protein 1 (KEAP1), Heme Oxygenase 1 (HO-1), and Superoxide Dismutase 1 (SOD1) in TDSCs treated with TBHP. Based on our results, Schottky heterojunction increased the total protein expression levels of BMAL1 and NRF2, as well as the downstream HO-1 and SOD1, while decreasing the expression level of KEAP1 in TDSCs (Fig. 5B,C). The immunofluorescence staining revealed an upregulation of BMAL1 and NRF2 expression in TDSCs within the TBHP + Schottky heterojunction group compared to the TBHP group. (Fig. 5D and Figure S7A). Immunofluorescence and immunohistochemical staining in vivo showed the same trend (Fig. 5E and Figure S7B-D). Given that NRF2 is sequestered by cytoplasmic KEAP1 and targeted for proteasomal degradation [38], we isolated nucleoproteins and cytoplasmic proteins to assess the expression of NRF2 and KEAP1 through Western blot analysis. The results demonstrated a significant upregulation of NRF2 expression in the nucleus upon stimulation with TBHP, which was further enhanced by Nb_2_C and Schottky heterojunction treatment. Notably, Schottky heterojunction exhibited superior efficacy in augmenting this increase in expression (Figure S7E,F). Moreover, KEAP1 expression in TDSCs exhibited a slight decreasing trend in the cytoplasm of the Schottky heterojunction + TBHP group, while no significant change was observed in the nucleus (Figure S7E,F). Furthermore, the immunofluorescence staining of TDSCs clearly showed higher density of NRF2 in the nucleus in the TBHP + Schottky heterojunction group compared to other groups (Fig. 5D and Figure S7A). In summary, these findings indicate that Schottky heterojunction exhibits enhanced capability in promoting the expression of *Bmal1*, thereby surpassing Nb_2_C in facilitating the expression and nuclear translocation of NRF2. Consequently, Schottky heterojunction shows promise as a therapeutic approach to treat AT by enhancing *Bmal1* expression.

### Schottky heterojunction ameliorate tendon deterioration in Achilles tendinopathy

To evaluate the therapeutic efficacy of the *Bmal1* enhancer Schottky heterojunction in treating AT, we compared it with the pure ROS scavenger Nb_2_C as the control group. Firstly, the results from the CCK-8 assay showed that both Nb_2_C and Schottky heterojunction significantly reduced the viability decrease of TDSCs induced by TBHP (Figure S8A), as supported by the live/dead staining results (Figure S8B). Furthermore, compared to the use of the ROS scavenger Nb_2_C, oxidative stress products such as ROS (Figure S8C,D), NADPH oxidase 1 (NOX1), and NOX4 were significantly reduced after the upregulation of *Bmal1* by Schottky heterojunction (Fig. 5F,G). Additionally, Schottky heterojunction exhibited stronger therapeutic effects in various aspects of Achilles tendon disease characteristics, including increased expression of COL3, MMP3, and MMP13 proteins (Fig. 5F,G), and increased mRNA expression of *Tnf-α*, IL-1, *IL-6*, and *Mmp3* (Fig. 5H). Moreover, consistent with the in vitro experiments, the results from in vivo experiments in rat AT showed that the Schottky heterojunction group had a more pronounced decrease in protein expression of COL3, MMP3, and MMP13 compared to the Nb_2_C group (Fig. 5I,J). Additionally, the mRNA levels of *Tnf-α*, *IL-6*, and *Mmp3* showed the same trend (Fig. 5K). Furthermore, both Nb_2_C and Schottky heterojunction treatments significantly increased the expression of COL1 (Fig. 5F-K). The histological analysis also demonstrated the superior therapeutic effect of the *Bmal1* enhancer Schottky heterojunction in rat Achilles tendon disease (Fig. 5L and Figure S8E,F). These in vitro and in vivo experimental results indicate that using Schottky heterojunction to upregulate *Bmal1* yields superior therapeutic effects for AT compared to using pure ROS scavenger Nb_2_C. Furthermore, the utilization of *Bmal1* enhancers represents a promising and potential treatment modality for AT.

### Schottky heterojunction exerts therapeutic effects by modulating the *Bmal1*-regulated *Nrf2*-mediated antioxidant stress mechanism

**Fig. 6:**
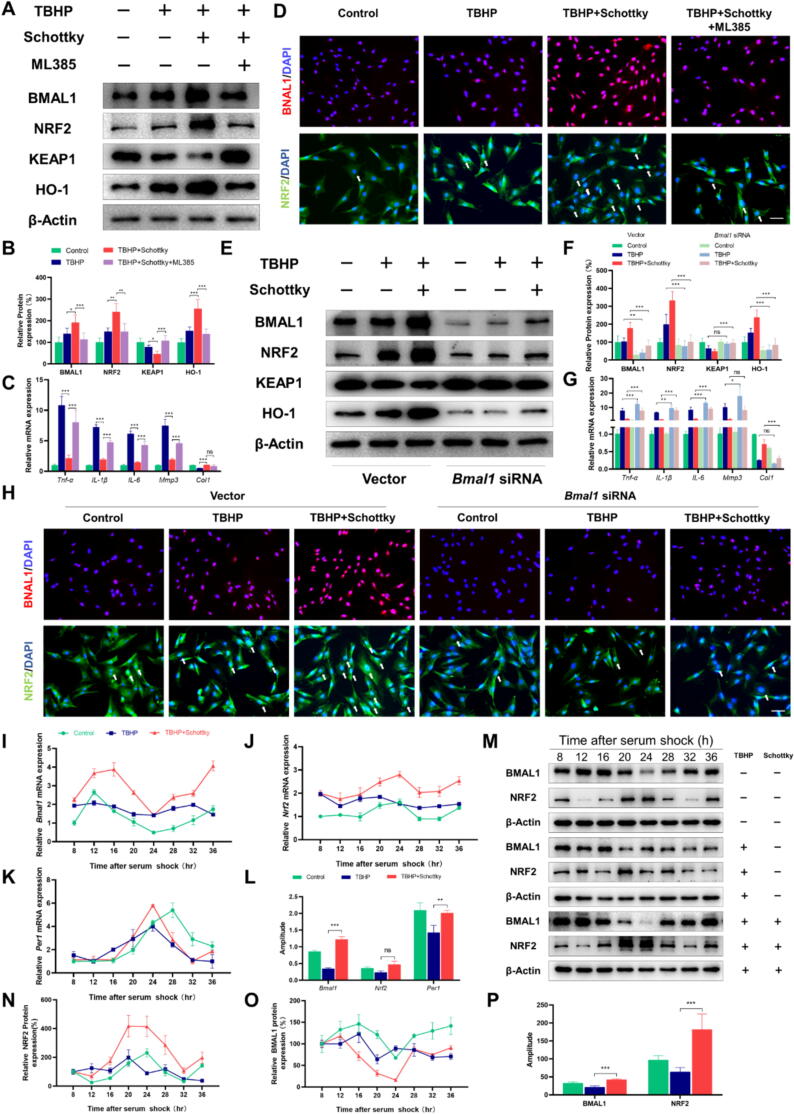
Schottky heterojunction exerts therapeutic effects by regulating the circadian clock in Achilles tendons and subsequently modulating the *Bmal1*-regulated *Nrf2*-mediated antioxidant stress mechanism. (A and B) Western blot analysis for BMAL1, NRF2, KEAP1, and HO-1 in TDSCs after TBHP treatment, treatment with or without Schottky heterojunction treatment, and treatment with or without ML385 (an *Nrf2* inhibitor). (C) Quantitative real-time PCR analysis of *Tnf-α*, *IL-6*, *Mmp3,* and *Col1* mRNA levels in the four groups. (D) Immunofluorescence staining for BMAL1 and NRF2 in the four groups. The white arrow indicates NRF2 in the nucleus. (E and F) Western blot analysis for BMAL1, NRF2, KEAP1, and HO-1 in TDSCs transfected with *Bmal1*-siRNA after TBHP treatment and treatment with or without Schottky heterojunction. (G) Quantitative real-time PCR analysis of *Tnf-α*, *IL-6*, *Mmp3,* and COL1 mRNA levels in the six groups. (H) Immunofluorescence staining for BMAL1 and NRF2 in the six groups. The white arrow indicates NRF2 in the nucleus. (I to K) The mRNA expression of *Bmal1*, *Nrf2,* and *Per1* at 4-hour intervals over a 28-hour period in TDSCs treated with or without TBHP after treatment with or without Schottky heterojunction. (L) The amplitude of the mRNA rhythmic expression of *Bmal1*, *Nrf2,* and *Per1* in the three groups. (M to O) The western blot analysis of BMAL1 and NRF2 at 4-hour intervals over a 28-hour period in TDSCs in the three groups. (P) The amplitude of BMAL1 and NRF2 protein rhythmic expression in the three groups. Data are presented as the mean ± SD. *p < 0.05, **p < 0.01, ***p < 0.001; ns, not significant. Scale bar, 100 μm.

To further elucidate whether the Schottky heterojunction exerts its therapeutic effects by modulating the *Nrf2*-mediated antioxidant stress mechanism regulated by *Bmal1*, we treated TDSCs separately with the *Nrf2* inhibitor ML385 and targeted *Bmal1* siRNA transfection. The ML385 molecule was employed as a specific probe to bind to Neh1, the cap “n” collar basic leucine zip domain of Nrf2, thereby impeding the binding of the V-Maf muscle-neurofibrosarcoma oncogene homologous G (MAFG)-NRF2 protein complex to regulatory DNA binding sequences[39]. Our investigation revealed that Schottky heterojunction's ability to rescue the decline in cell viability caused by TBHP (Figure S9A) and the ROS scavenging ability (Figure S9B,C) were inhibited by the addition of ML385. Furthermore, Western blotting demonstrated that Schottky heterojunction, in combination with TBHP, increased the total protein expression of BMAL1, NRF2, and HO-1 compared to TBHP alone, but ML385 significantly suppressed this effect, while KEAP1 levels were increased by ML385 (Fig. 6**A,B**). In addition, immunofluorescence staining of TDSCs corroborated the Western blotting results (Fig. 6D and Figure S9D,E). Furthermore, the qPCR analysis revealed that ML385 compromised the inhibitory effect of Schottky heterojunction on *Tnf-α*, *IL-1β*, *IL-6*, and *Mmp3* expression while also attenuating its promoting effect on *Col1* expression (Fig. 6C). Next, we transfected siRNA against *Bmal1* into TDSCs under oxidative stress in the presence or absence of Schottky heterojunction. As the CCK-8 results show, Schottky heterojunction's ability to reverse the TBHP-induced decline in cell viability was attenuated after transfection with siRNA (Figure S9F). Furthermore, the ROS scavenging ability of Schottky heterojunction was also inhibited by *Bmal1* knockdown (Figure S9G,H). The Schottky heterojunction’s ability to increase BMAL1, NRF2 and HO-1 expression was partially inhibited after siRNA transfection (Fig. 6E,F), and immunofluorescence staining of TDSCs were supported by the Western blotting results (Fig. 6H and Figure S9I,J). Additionally, the effects of Schottky heterojunction on *Tnf-α*, *IL-1β*, *IL-6*, *Mmp3,* and *Col1* expression were inhibited by *Bmal1* knockdown (Fig. 6G).

Furthermore, the results of the mRNA oscillations of key circadian components in TDSCs following Schottky heterojunction treatment revealed compelling results. Pre-treatment with Schottky heterojunction reversed the reduced amplitude of daily oscillations of *Bmal1*, *Nrf2*, and *Per1* induced by TBHP (Fig. 6I-L and Figure S9K). Consistent with the real-time quantitative PCR analysis, protein immunoblotting analysis revealed a significant decrease in the oscillation amplitudes of BMAL1 and NRF2 expression levels after TBHP treatment (Fig. 6M-P and Figure S9L). Furthermore, rescue experiments demonstrated a significant increase in the oscillation amplitudes of BMAL1 and NRF2 protein levels following Schottky heterojunction treatment (Fig. 6M,P). These findings suggest that Schottky heterojunction potentially exerts therapeutic effects by augmenting the amplitude of circadian rhythmic expression, thereby enhancing the *Bmal1*-regulated *Nrf2*-mediated antioxidant stress mechanism.

### Different administration time affects the efficacy of Achilles tendinopathy in rats

**Fig. 7:**
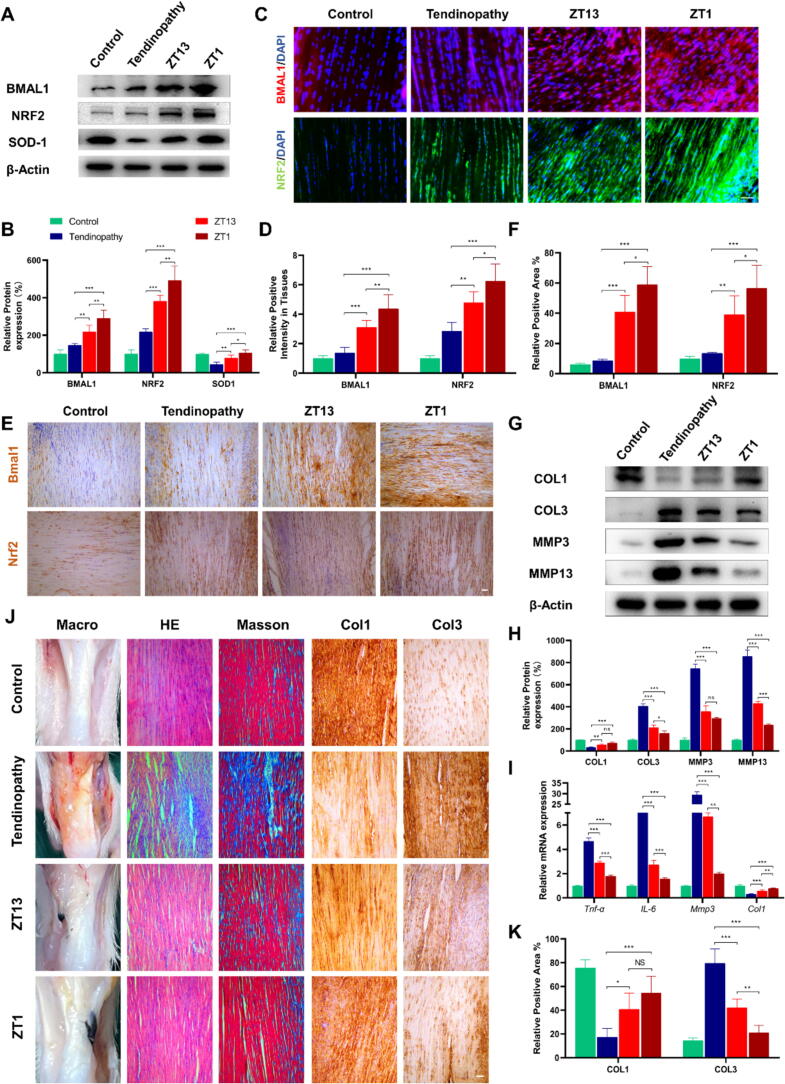
Different administration time affects the efficacy of Achilles tendinitis in rats. (A and B) Western blot analysis for BMAL1, NRF2, and SOD-1 in the rat tendon tissues of rats in the four groups (control, tendinopathy, ZT13, and ZT1). (C and D) Immunofluorescence staining for BMAL1 and NRF2 in rat Achilles tendon tissues in the four groups. (E and F) immunohistochemistry staining for BMAL1 and NRF2 in rat Achilles tendon tissues in the four groups. (G and H) Western blot analysis for COL1, COL3, MMP3 and MMP13 in rat Achilles tendon tissues in the four groups. (I) Quantitative real-time PCR analysis of *Tnf-α*, *IL-1β*, *IL-6*, *Mmp3,* and *Col1* mRNA levels in rat Achilles tendon tissues in the four groups. (J and K) Macro-picture, HE staining of tendon, Masson staining, and immunohistochemistry of COL1 and COL3 in the tendon tissues of rats in the four groups. Data are presented as the mean ± SD. *p < 0.05, **p < 0.01, ***p < 0.001; ns, not significant. Scale bar, 100 μm.

Our above studies have shown that the expression of core clock genes in tendon tissues exhibits a circadian rhythm and enhancing the amplitude of circadian rhythmicity expression strengthens its stability. These findings provide us with a novel insight that by adjusting the administration timing, we may be able to maximize the therapeutic efficacy of *Bmal1* enhancer Schottky heterojunction. Based on our observations that *Bmal1* and *Nrf2* exhibit troughs at approximately ZT13 and peaks at ZT1, while *IL-6* and *Mmp3* also follow similar timing patterns, we conducted an experiment to investigate the timing of administration. Specifically, the model organism rats were divided into four groups, including a control group, tendinopathy group, Schottky heterojunction (ZT13) group, and Schottky heterojunction (ZT1) group. Achilles tendinitis was induced using established methods, and each rat in the Schottky heterojunction ZT13 and ZT1 groups received a 50 μl injection of Schottky heterojunction (80 mg/ml) at the left Achilles bone-tendon junction, either at ZT13 or ZT1, respectively. Western blot analysis showed that injection of Schottky heterojunction at the ZT1 time point induced higher BMAL1, SOD-1 and NRF2 expression than injection at the ZT13 time point (Fig. 7**A,B**). This was also confirmed by tissue immunofluorescence staining and tissue immunochemical staining (Fig. 7C-F). Additionally, injection at the ZT1 time point exhibited stronger therapeutic effects in various aspects of Achilles tendon disease characteristics, including decreased expression of COL1 (Fig. 7G-I), increased expression of COL3, MMP3, and MMP13 proteins (Fig. 7G,H), and increased mRNA expression of *Tnf-α*, *IL-6*, and *Mmp3* (Fig. 7I). Moreover, histological analysis also demonstrated the superior therapeutic effect of the injection at ZT1 time point in rat Achilles tendon disease (Fig. 7J,K). These data thus revealed that injection of Schottky heterojunction at the time point of ZT1 has a better effect than injection at ZT13, that is, enhancement of *Bmal1* expression at the peak of expression of *Bmal1*, *Nrf2*, *IL-6,* and *Mmp3* has a better therapeutic effect than at the trough.

## Discussion

The mammalian circadian clock is a coordinated network of hierarchical oscillators [40]. The central clock can transmit temporal cues to peripheral tissues and influence their circadian rhythms through neurohumoral regulation [41]. Prior research has demonstrated the presence of core clock proteins in peripheral tissues, and various organs, including the liver, gut, pancreas, bone, and intervertebral discs, exhibit intrinsic clock rhythms as peripheral oscillators [22], [42], [43]. Nevertheless, circadian rhythms in the Achilles tendon remain unexplored. In this study, we demonstrated for the first time that the Achilles tendon is a peripheral oscillator and that the inner circadian clock may play an important role in the development of AT.

Various etiologies of AT have been described, including mechanical, inflammatory, and oxidative stress theories [3]. However, these theories have not fully elucidated AT's pathogenesis [44]. Circadian misalignment ubiquitously exists and is associated with dysrhythmia in numerous diseases [22], [45]. Nevertheless, the role of circadian clock dysrhythmia in AT remains inadequately investigated. In this study, we found for the first time that AT significantly impairs clock system accuracy and robustness. Disruption of local circadian rhythm in the Achilles tendon serves as the fundamental pathological event, which in turn aggravates the tissue damage of the Achilles tendon.

The core protein BMAL1 is thought to be essential for coordinating clock output and metabolic adaptation, playing a vital defense against infection, ROS accumulation, and oxidative damage [46]. Our study provided confirmation of the protective role of *Bmal1* in the Achilles tendon. Specifically, we observed that the knockdown of *Bmal1* disrupted the circadian clock in the Achilles tendon and exacerbated inflammation. Conversely, up-regulation of *Bmal1* demonstrated a protective effect. This protective effect was mediated by controlling the expression of *Nrf2* and its protective antioxidant response in the Achilles tendon. Our previous study found that *Nrf2* is activated in response to oxidative stress in the Achilles tendon, and the inhibition of *Nrf2* leads to more severe Achilles tendon injury [32]. Furthermore, BMAL1 has been demonstrated to induce *Nrf2* transcription in certain tissues through its binding to an E-box element within the *Nrf2* promoter, thereby facilitating the upregulation of antioxidant genes [17], [47]. Interestingly, Early et al. observed that the rhythmic expression of BMAL1 and NRF2 is synchronized in peritoneal cells in vivo but desynchronized in bone marrow-derived macrophages in vitro[17]. This finding is analogous to our research on Achilles tendon. We hypothesize that in vitro, *Nrf2* may be regulated by BMAL1, similar to other circadian rhythm genes controlled by BMAL1, such as *Per1*, and *Per2*. In fact, these circadian rhythm genes controlled by BMAL1 also exhibit expression patterns that are not synchronized with BMAL1. Therefore, it is possible that the rhythmic expression of BMAL1 and NRF2 is desynchronized in vitro. The synchronized rhythmic expression of BMAL1 and NRF2 in vivo may be influenced by the central clock or other factors, and the specific mechanisms require further in-depth investigation. Nonetheless, our study revealed a direct transcriptional regulation of the *Nrf2* gene by *Bmal1* in TDSCs. Here, we describe a mechanism in which oxidative stress and inflammation in Achilles tendinopathy trigger early reactive expression of *Bmal1* and *Nrf2* as a defense response to external stimuli. Simultaneously, these stimuli reduce the amplitude of rhythmic expression of *Bmal1* and *Nrf2*, disrupting the circadian clock pattern [6]. As a result, the *Nrf2*-mediated antioxidant and anti-inflammatory defense mechanism regulated by the circadian clock is compromised, leading to decreased *Bmal1* and *Nrf2* levels in the advanced stage of AT, culminating in the progression of the disease. Active overexpression of *Bmal1* increases the amplitude and absolute value of rhythmic expression of *Bmal1* and *Nrf2*. A rhythmic expression pattern with high amplitude is known to be more resilient to external interference [48], [49], thereby enhancing the *Nrf2*-mediated antioxidant defense mechanism and alleviating tendon injury.

Treatment of diseases by targeting the circadian clock has been studied in other organs and tissue [50], [51]. However, there remains a significant lack of pharmacological agents capable of directly and efficiently regulating the expression of *Bmal1*. The BMAL1 is a protein with a PAS domain [26], which can serve as a sensor for REDOX potential, oxygen, cellular energy, or light [27]. Previous studies have shown that PAS domains are expressed in voltage-gated channels and that truncation of PAS domains will inactivate the voltage-gated channel [52]. In addition, K + currents are closely related to the expression of neurophysiological rhythms, and acute inhibition of large-conductance calcium-activated potassium channel (BK) currents blunts the spike frequency of circadian SCN neurons [53]. Given these results, we began to explore the potential that PAS domains in clock proteins might transduce not only optical signals but also electrical signals [54]. This led us to consider the possibility of modulating the circadian clock through intracellular electrical signals as a potential therapeutic approach for treating AT, as various environmental cues have been known to regulate the circadian clock [54], [55]. Schottky heterojunction possesses the efficacy on regulating and/or amplifying bioelectrical signals through the directional carrier migration from semiconductor to metal matrix [56], which is brought from the Schottky interfacial barrier [57]. In this study, we synthesized a novel material with Schottky heterojunction to treat AT by modulating the expression of *Bmal1* through intercellular electrical signaling. DFT calculation, especially HSE06 package, was combined with its photoelectric performance to demonstrate the modulating and/or amplifying electrical signals upon Schottky heterojunction. It can be attributed to the carrier directional transfer from semiconductor to metal. Moreover, two factors that might affect the composition and mechanism of Schottky heterojunction were considered: (Ⅰ) The Schottky model had been updated to consider the REDOX reactions that occurred in the humoral environment. In this situation, the Schottky heterojunction can still modulate and amplify electrical signals, which means that the electrical modulation is stable and durable in vivo/vitro. (Ⅱ) In our previous study, we found that Nb_2_C nanosheets with oxygen-rich edge defects have the efficacy of removing ROS [28], which can be attributed to the physical adsorption capacity of Nb_2_C nanosheets. Therefore, we compare the Schottky heterojunction and Nb_2_C to more strictly reflect the electrical signals’ regulation assigned by Schottky heterojunction. Although we did not extensively investigate changes in intracellular electrical signaling, we were encouraged by the clear therapeutic effect of Schottky heterojunction on AT. The treatment mechanism of Schottky heterojunction for AT can be attributed to the “triple hit” approach: (I) enhancing the expression of *Bmal1* and *Nrf2* to bolster the body's antioxidant and anti-inflammatory defense mechanism; (II) increasing the amplitude of rhythmic expression of *Bmal1* and *Nrf2* to restore stable circuitry and clock output; and (III) directly adsorbing and scavenging ROS to mitigate ROS-induced oxidative stress damage. Our study demonstrated that, compared to the ROS scavenger Nb_2_C alone, Schottky heterojunction could act as a *Bmal1* enhancer, regulating the *Nrf2*-mediated anti-oxidative stress defense mechanism and yielding superior therapeutic effects.

However, current treatments for AT mostly ignore the timing of drug intake, which is chosen based on practicality or attempts to eliminate side effects during the active phase of the patient [58], [59], [60]. Studies have shown that administering medication at the peak and trough of circadian rhythm expression yields distinct effects [21], [61]. For instance, the severity of pulmonary fibrosis is highly contingent on treatment timing and inversely correlated with NRF2 protein levels. Administering bleomycin near the peak or nadir of NRF2 protein expression (ZT0 or ZT12, respectively) results in contrasting outcomes, with significantly higher fibrosis scores observed at ZT12 [21]. Similarly, treating obese mice with NAD + revealed that the response varied when given one hour before the NAD + peak and trough, respectively. The latter failed to adequately reflect improvements in metabolic parameters [61]. These findings strongly support that circadian rhythms initiate time-dependent tissue-protective responses to various diseases. Similarly, our study identified a treatment time dependence, showing that injecting Schottky heterojunction at the peak of *Bmal1*, *Nrf2*, *IL6* and *Mmp3* expression yielded greater effectiveness than at the trough. We propose that this may be due to increased amplitude of *Bmal1* and *Nrf2* expression when injected at their respective peaks, as well as the suppression of *IL6* and *Mmp3* expression at their peaks. Our findings underscore the critical significance of considering circadian rhythms in therapeutic development, emphasizing the need for precise treatment timing to optimize the potential of *Bmal1*-based therapies. These insights contribute to the advancement of chronobiological research and pave the way for innovative therapeutic approaches targeting circadian processes.

However, our study has several limitations. Firstly, a larger number of Achilles tendon sample is needed to understand *Bmal1* expression changes in AT patients at different stages. Secondly, this study focused solely on prolonged darkness as a circadian clock disorder, while real-life scenarios such as shift work hold greater clinical significance. Further research is warranted to investigate the impact of circadian rhythm disorders, such as those induced by jet lag, on AT. Additionally, to elucidate the role of circadian rhythms in the pathogenesis of AT, it is necessary to establish AT models in *Bmal1* knockout mice or other clock gene knockout mice, such as *Nr1d1* knockout mice. Finally, investigating how Schottky heterojunctions regulate *Bmal1* expression via intercellular electrical signals requires further exploration, potentially facilitating the design of precise circadian rhythm regulators.

## Conclusion

In conclusion, our study elucidates the protective function of the intact circadian clock in the Achilles tendon, emphasizing the critical function of *Bmal1* in the onset and advancement of AT through the regulation of *Nrf2*-mediated antioxidant and anti-inflammatory pathways. Enhancing the expression and rhythmic amplitude of *Bmal1* through interface Schottky barrier, in conjunction with strategically timing its administration, holds immense promise for advancing the future treatment of AT and the development of innovative therapeutic approaches targeting circadian rhythms.

## Ethics statement

All experiments involving animals were conducted according to the ethical policies and procedures approved by the Ethics Committee and the Institutional Animal Care and Use Committee of Nanjing Drum Tower Hospital, Nanjing University Medical School, China (2022AE02002). All experiments involving tissue samples from human were conducted according to the ethical policies and procedures approved by the ethics committee of Nanjing Drum Tower Hospital, Nanjing University Medical School, China (2020–156-01).

## Declaration of competing interest

The authors declare that they have no known competing financial interests or personal relationships that could have appeared to influence the work reported in this paper.

## References

[1] de Jonge S., van den Berg C., de Vos R., van der Heide H., Weir A., Verhaar J. (2011). Incidence of midportion Achilles tendinopathy in the general population. Br J Sports Med.

[2] Riley G. (2008). Tendinopathy–from basic science to treatment. Nat Clin Pract Rheumatol.

[3] Magnusson S., Langberg H., Kjaer M. (2010). The pathogenesis of tendinopathy: balancing the response to loading, Nature reviews. Rheumatology.

[4] Zhang S., Ju W., Chen X., Zhao Y., Feng L., Yin Z. (2022). Hierarchical ultrastructure: An overview of what is known about tendons and future perspective for tendon engineering. Bioact Mater.

[5] Millar N., Murrell G., McInnes I. (2017). Inflammatory mechanisms in tendinopathy - towards translation. Nat Rev Rheumatol.

[6] Musiek E., Lim M., Yang G., Bauer A., Qi L., Lee Y. (2013). Circadian clock proteins regulate neuronal redox homeostasis and neurodegeneration. J Clin Invest.

[7] Lee J., Moulik M., Fang Z., Saha P., Zou F., Xu Y. (2013). Bmal1 and β-cell clock are required for adaptation to circadian disruption, and their loss of function leads to oxidative stress-induced β-cell failure in mice. Mol Cell Biol.

[8] Abdel-Rahman E., Hosseiny S., Aaliya A., Adel M., Yasseen B., Al-Okda A. (2021). Sleep/wake calcium dynamics, respiratory function, and ROS production in cardiac mitochondria. J Adv Res.

[9] Early J., Curtis A. (2016). Immunometabolism: Is it under the eye of the clock?. Semin Immunol.

[10] Wu J., Jing X., Du Q., Sun X., Holgersson K., Gao J. (2023). Disruption of the Clock Component Bmal1 in Mice Promotes Cancer Metastasis through the PAI-1-TGF-β-myoCAF-Dependent Mechanism. Adv Sci.

[11] Xin M., Bi F., Wang C., Huang Y., Xu Y., Liang S. (2024). The circadian rhythm: A new target of natural products that can protect against diseases of the metabolic system, cardiovascular system, and nervous system. J Adv Res.

[12] Reppert S., Weaver D. (2002). Coordination of circadian timing in mammals. Nature.

[13] Roenneberg T., Merrow M. (2005). Circadian clocks - the fall and rise of physiology, Nature reviews. Mol Cell Biol.

[14] Bunger M., Wilsbacher L., Moran S., Clendenin C., Radcliffe L., Hogenesch J. (2000). Mop3 is an essential component of the master circadian pacemaker in mammals. Cell.

[15] Takahashi J. (2017). Transcriptional architecture of the mammalian circadian clock, Nature reviews. Genetics.

[16] Li R., Xiao J., Cao Y., Huang Q., Ho C.-T., Lu M. (2022). Capsaicin Attenuates Oleic Acid-Induced Lipid Accumulation via the Regulation of Circadian Clock Genes in HepG2 Cells. J Agric Food Chem.

[17] Early J., Menon D., Wyse C., Cervantes-Silva M., Zaslona Z., Carroll R. (2018). Circadian clock protein BMAL1 regulates IL-1β in macrophages via NRF2. PNAS.

[18] Lu Z., Zhao R., Li Y., Wang J., Guo J., Bai C. (2024). Smart antioxidant function enhancing (SAFE) nucleic acid therapy for ROS-related chronic diseases and comorbidities. Bioact Mater.

[19] Mills E.L., Ryan D.G., Prag H.A., Dikovskaya D., Menon D., Zaslona Z. (2018). Itaconate is an anti-inflammatory metabolite that activates Nrf2 via alkylation of KEAP1. Nature.

[20] Zhang Q., Liu J., Duan H., Li R., Peng W., Wu C. (2021). viaActivation of Nrf2/HO-1 signaling: An important molecular mechanism of herbal medicine in the treatment of atherosclerosis the protection of vascular endothelial cells from oxidative stress. J Adv Res.

[21] Pekovic-Vaughan V., Gibbs J., Yoshitane H., Yang N., Pathiranage D., Guo B. (2014). The circadian clock regulates rhythmic activation of the NRF2/glutathione-mediated antioxidant defense pathway to modulate pulmonary fibrosis. Genes Dev.

[22] W. Jiang, L. Jin, D. Ju, Z. Lu, C. Wang, X. Guo, H. Zhao, S. Shen, Z. Cheng, J. Shen, G. Zong, J. Chen, K. Li, L. Yang, Z. Zhang, Y. Feng, J. Shen, E. Zhang, R. Wan, The pancreatic clock is a key determinant of pancreatic fibrosis progression and exocrine dysfunction, Science translational medicine 14(664) (2022) eabn3586. 10.1126/scitranslmed.abn3586 36170444

[23] Tohidnezhad M., Varoga D., Wruck C., Brandenburg L., Seekamp A., Shakibaei M. (2011). Platelet-released growth factors can accelerate tenocyte proliferation and activate the anti-oxidant response element. Histochem Cell Biol.

[24] Xie M., Tang Q., Nie J., Zhang C., Zhou X., Yu S. (2020). Porphyromonas GingivalisBMAL1-Downregulation Aggravates -Induced Atherosclerosis by Encouraging Oxidative Stress. Circ Res.

[25] Cardinali D., Srinivasan V., Brzezinski A., Brown G. (2012). Melatonin and its analogs in insomnia and depression. J Pineal Res.

[26] Zhulin I., Taylor B., Dixon R. (1997). PAS domain S-boxes in Archaea, Bacteria and sensors for oxygen and redox. Trends Biochem Sci.

[27] Taylor B., Zhulin I. (1999). PAS domains: internal sensors of oxygen, redox potential, and light. Microbiol Mol Biol Rev.

[28] Sun K., Wu Y., Xu J., Xiong W., Xu W., Li J. (2022). Niobium carbide (MXene) reduces UHMWPE particle-induced osteolysis. Bioact Mater.

[29] Ren X., Huo M., Wang M., Lin H., Zhang X., Yin J. (2019). Highly Catalytic Niobium Carbide (MXene) Promotes Hematopoietic Recovery after Radiation by Free Radical Scavenging. ACS Nano.

[30] Bao S., Yu D., Tang Z., Wu H., Zhang H., Wang N. (2024). Conformationally regulated “nanozyme-like” cerium oxide with multiple free radical scavenging activities for osteoimmunology modulation and vascularized osseointegration. Bioact Mater.

[31] Wu Y., Song X., Zhou X., Song R., Tang W., Yang D. (2023). Piezo-Activated Atomic-Thin Molybdenum Disulfide/MXene Nanoenzyme for Integrated and Efficient Tumor Therapy via Ultrasound-Triggered Schottky Electric Field. Small.

[32] Xu X., Wang R., Li Y., Wu R., Yan W., Zhao S. (2023). Cerium oxide nanozymes alleviate oxidative stress in tenocytes for Achilles tendinopathy healing. Nano Res.

[33] Balsalobre A., Damiola F., Schibler U. (1998). A serum shock induces circadian gene expression in mammalian tissue culture cells. Cell.

[34] Schwartz W., Zimmerman P. (1990). Circadian timekeeping in BALB/c and C57BL/6 inbred mouse strains. J Neurosci.

[35] Oshita T., Tobita M., Tajima S., Mizuno H. (2016). Adipose-Derived Stem Cells Improve Collagenase-Induced Tendinopathy in a Rat Model. Am J Sports Med.

[36] Ramkisoensing A., Gu C., van Engeldorp Gastelaars H., Michel S., Deboer T., Rohling J. (2014). Enhanced phase resetting in the synchronized suprachiasmatic nucleus network. J Biol Rhythms.

[37] Tan H., Kong P., Zhang R., Gao M., Liu M., Gu X. (2021). Controllable Generation of Reactive Oxygen Species on Cyano-Group-Modified Carbon Nitride for Selective Epoxidation of Styrene. Innovation (Cambridge (Mass)).

[38] Cuadrado A., Rojo A., Wells G., Hayes J., Cousin S., Rumsey W. (2019). Therapeutic targeting of the NRF2 and KEAP1 partnership in chronic diseases. Nat Rev Drug Discov.

[39] Wheeler M.A., Clark I.C., Tjon E.C., Li Z., Zandee S.E.J., Couturier C.P. (2020). MAFG-driven astrocytes promote CNS inflammation. Nature.

[40] M. Jennifer A, G. Carla B, T. Joseph S, Central and peripheral circadian clocks in mammals, Annu Rev Neurosci 35(0) (2012). 10.1146/annurev-neuro-060909-153128 PMC3710582 22483041

[41] N. Khoa D, F. Sarah J, Q. Yifu, Y. Karen, C. Jeffery S, C. Ajay, Circadian gene Bmal1 regulates diurnal oscillations of Ly6C(hi) inflammatory monocytes, Science 341(6153) (2013). 10.1126/science.1240636 PMC3836670 23970558

[42] Dong W., Pandi P., Michal D., Xueyu H., Xiaolong X., Qiliang S. (2022). Restoring the dampened expression of the core clock molecule BMAL1 protects against compression-induced intervertebral disc degeneration. Bone Res.

[43] M. Cameron S, S. Filip K, Circadian Influence on Metabolism and Inflammation in Atherosclerosis, Circ Res 119(1) (2016). 10.1161/CIRCRESAHA.116.308034 PMC4922503 27340272

[44] Dong Z., Peng R., Zhang Y., Shan Y., Ding W., Liu Y. (2023). Tendon Repair and Regeneration Using Bioinspired Fibrillation Engineering That Mimicked the Structure and Mechanics of Natural Tissue. ACS Nano.

[45] Kolarski D., Sugiyama A., Breton G., Rakers C., Ono D., Schulte A. (2019). Controlling the Circadian Clock with High Temporal Resolution through Photodosing. J Am Chem Soc.

[46] Kondratov R. (2007). A role of the circadian system and circadian proteins in aging. Ageing Res Rev.

[47] Zhu M., Tang H., Tang X., Ma X., Guo D., Chen F. (2018). BMAL1 suppresses ROS-induced endothelial-to-mesenchymal transition and atherosclerosis plaque progression via BMP signaling. Am J Transl Res.

[48] O'Neill J., Reddy A. (2011). Circadian clocks in human red blood cells. Nature.

[49] Pinato D., Stebbing J. (2016). Melatonin: resetting the clock of cancer progression?. Lancet Oncol.

[50] Qi G., Wu W., Mi Y., Shi R., Sun K., Li R. (2018). Tea polyphenols direct Bmal1-driven ameliorating of the redox imbalance and mitochondrial dysfunction in hepatocytes. Food Chem Toxicol.

[51] Zhao Y., Xu L., Ding S., Lin N., Ji Q., Gao L. (2017). Novel protective role of the circadian nuclear receptor retinoic acid-related orphan receptor-α in diabetic cardiomyopathy. J Pineal Res.

[52] Malak O., Gluhov G., Grizel A., Kudryashova K., Sokolova O., Loussouarn G. (2019). Voltage-dependent activation in EAG channels follows a ligand-receptor rather than a mechanical-lever mechanism. J Biol Chem.

[53] Pitts G., Ohta H., McMahon D. (2006). Daily rhythmicity of large-conductance Ca2+ -activated K+ currents in suprachiasmatic nucleus neurons. Brain Res.

[54] Sidote D., Majercak J., Parikh V., Edery I. (1998). Differential effects of light and heat on the Drosophila circadian clock proteins PER and TIM. Mol Cell Biol.

[55] Jones J., Tackenberg M., McMahon D. (2015). Manipulating circadian clock neuron firing rate resets molecular circadian rhythms and behavior. Nat Neurosci.

[56] Muhammad F., Tahir M., Zeb M., Kalasad M., Mohd Said S., Sarker M. (2020). Synergistic enhancement in the microelectronic properties of poly-(dioctylfluorene) based Schottky devices by CdSe quantum dots. Sci Rep.

[57] Liu C., Ma W., Chen M., Ren W., Sun D. (2019). A vertical silicon-graphene-germanium transistor. Nat Commun.

[58] Münch M., Kramer A. (2019). Timing matters: New tools for personalized chronomedicine and circadian health. Acta Physiol (Oxf).

[59] Ruben M., Smith D., FitzGerald G., Hogenesch J. (2019). Dosing time matters. Science (New York, NY).

[60] Peeples L. (2018). Medicine's secret ingredient - it's in the timing. Nature.

[61] E.-C. Quetzalcoatl, M.-V. Lucía, G.-S. Mirna, S.-O. Román, V.-V. Laura A, B.-P. Fernando, P.-B. Ignacio, C.-V. Erick, M.-S. Paola, B.-Z. Marcia, O.-S. Ricardo, A.-A. Lorena, Time-of-day defines NAD(+) efficacy to treat diet-induced metabolic disease by synchronizing the hepatic clock in mice, Nat Commun 14(1) (2023). 10.1038/s41467-023-37286-2 PMC10043291 36973248

