# Supplementary material for: Achilles tendinopathy treatment via circadian rhythm regulation
Source: J Adv Res. 2024 Oct 21;75:505–20. doi: 10.1016/j.jare.2024.10.022 (PMC12789736; doi:10.1016/j.jare.2024.10.022)
Supplement: Supplementary Data 1 [file mmc1.docx]

**Supporting Information for**

Achilles tendinopathy treatment via circadian rhythm regulation

**This PDF file includes:**

Supporting text

Figures S1 to S9

Tables S1 to S2

Supporting Information Text

**Materials and Methods**

**Animal Studies for Zeitgeber Time (ZT)**

The Sprague Dawley (SD) male rats (8 weeks old), obtained from the Animal Center of Nanjing Medical University (Jiangsu, China), were housed in a controlled environment with stable temperature and humidity under a 12/12 h light/dark cycle (LD) (lights on at 8:00 a.m.; lights off at 8:00 p.m., where ZT0 and ZT12 represent the times when lights were switched on and off, respectively). For the light deprivation protocol resulting in constant darkness (DD), eight-week-old male SD rats were kept in complete darkness.SD male rats were euthanized by cervical dislocation, and tendons were collected at specific time points (ZT1, ZT5, ZT9, ZT13, ZT17 and ZT21) for Real-time quantitative PCR analysis and Western Blot detection. Statistical analyses were conducted utilizing Origin 8 software (version 8.6, OriginLab Corporation, MA, USA). Data are expressed as means ± standard deviation (SD). Circadian variations, encompassing amplitude and period length, were determined by fitting a cosine-wave equation [y = Baseline + (Amplitude × Cos (x × 2 × π/Period + C))] to the clock gene expression data. All animal experiments were conducted strictly in accordance with the Guide for the Care and Use of Laboratory Animals and obtained approval from the Animal Investigation Ethics Committee of Nanjing Drum Tower Hospital.

**C****ell culture**

The tendon derived stem cells (TDSCs) were isolated and cultured as previously described. Briefly, the hind limb Achilles tendons dissected from euthanized Sprague−Dawley (SD) rats (3-4 weeks old, male) cut into 1 mm^3^ fragments and washed with PBS three times. Subsequently, the fragments were lysed with 3 mg/ml collagenase I (Sigma-Aldrich, USA) in DMEM (Gibco, USA) at 37℃ for 8 h. After stopping the digestion with 5 ml PBS and filtrating with a 200 μm mesh filter, the resulting suspension was centrifuged at 1500 rpm for 5 min. After undergoing filtration and centrifugation, the cells were resuspended in DMEM medium (Gibco, USA) supplemented with 1% penicillin-streptomycin solution (P/S, Gibco) and 10% fetal bovine serum (FBS, Gibco, USA) in a 100 mm dish. The cell culture was maintained at 37 ℃ in a humidified atmosphere containing 5% CO2, with the medium being refreshed every two days.

**Synchronization experiments with TDSCs**

TDSCs were treated with 50% horse serum (HyClone, Logan, UT, USA) for 2 hours as described in the previous paper. The medium was then replaced with DMEM medium supplemented with 1% penicillin-streptomycin solution and 10% fetal bovine serum. Subsequently, the protein and mRNA were extracted every 4 hours, 8-36 hours after serum shock, and detected by Real-time quantitative PCR and Western Blot. Statistical analyses were conducted utilizing Origin 8 software (version 8.6, OriginLab Corporation, MA, USA). Data are expressed as means ± standard deviation (SD). Circadian variations, encompassing amplitude and period length, were determined by fitting a cosine-wave equation [y = Baseline + (Amplitude × Cos (x × 2 × π/Period + C))] to the clock gene expression data.

**Human tendon samples**

Tendon tissues (n = 6) were obtained from patients who underwent Arthroscopic surgery and total knee replacement surgery with motor system injury including tendinopathy (n = 3) and normal tendon tissues obtained from amputated patients (n = 3) at Nanjing Drum Hospital. The study protocol received approval from the Ethics Committee of Nanjing Drum Hospital. Acquisition of human materials followed informed consent procedures, and all research activities adhered to the principles delineated in the Declaration of Helsinki.

**Establishment of AT Rats**

The Sprague Dawley (SD) male rats (8 weeks old), obtained from the Animal Center of Nanjing Medical University (Jiangsu, China), were randomly assigned to their respective treatment groups. After a one-week acclimation period with unrestricted access to food and water, a solution of collagenase I (Sigma-Aldrich) at a concentration of 5 mg/mL or saline (sham group) was injected into the left Achilles bone-tendon junction every 2 days at Zeitgeber Time 1 (ZT1) to induce tendon injury, except for the control group. Following a 14-day period, appropriate measures were taken. Subsequently, after an additional 14 days, at the time point of ZT1, tendons were extracted and prepared for HE staining, Masson staining, immunofluorescence staining, and immunohistochemical staining. Meanwhile, mRNA and proteins were extracted from the tendons in these groups and detected using Real-time quantitative PCR and Western Blot.

**Synthetic Procedures of materials**

Nb_2_AlC powder with a purity over 99% was acquired from Feynman Nano Technology Co., Ltd. We sourced Lithium Fluoride (LiF, 99.9% metals basis) and Tetrapropylammonium hydroxide (TPAOH) aqueous solution from Alfa Aesar Chemicals Co. Ltd. Additionally, Cerium nitrate hexahydrate (Ce(NO_3_)_3_·6H_2_O, 99.95%), ethylene glycol (AR, 98%), and ammonia solution (AR, 25–28%) were procured from Aladdin Co. Ltd. Hydrochloric acid (HCl, 36.5–38.0 wt%) came from Merck Pty. Ltd. These chemicals were used as received, without any additional purification.

The process of synthesizing bulk Nb_2_C nanosheets (NSs) started with chemical exfoliation of Nb_2_AlC. Initially, 1.6g of LiF was gradually dissolved in 20mL of 9M HCl, stirred for 5 minutes, and followed by the addition of 1g Nb_2_AlC over 10 minutes. The mixture was then stirred at 45°C for a day, followed by repetitive washing with deionized water and centrifugation at 3500 rpm for 5 minutes each, done 6-8 times until the pH exceeded 6.0. The resultant sediment was dried overnight at 60°C in a vacuum.

For further processing, 150 mg of the obtained bulk Nb_2_C was mixed with 100 mL TPAOH and stirred at room temperature for 72 hours. This mixture was filtered through a 0.22 μm membrane, repeatedly washed with deionized water, and then freeze-dried.

To prepare a uniform suspension, 100 mg of the few-layer Nb_2_C nanosheets were stirred vigorously in 10 mL aqueous solution for 2 hours, achieving a concentration of approximately 10 mg/mL. Then, a solution of 5 mL ethylene glycol containing 12.6 mg of Ce(NO_3_)_3_·6H_2_O was gradually added to 10 mL of the Nb_2_C suspension, continuously stirred for 5 minutes. The mixture was then heated in a water bath at 60 °C with continuous stirring. After 10 minutes, 100 μL of ammonia water (28–30%) was injected rapidly using a syringe. Following 2 hours of stirring, the resultant suspension was centrifuged and washed until neutral pH was achieved. The final product, designated as Nb_2_C@CeO_2_ (Schottky heterojunction), was either re-dispersed in water or freeze-dried for subsequent use.

**Characterization of materials**

Scanning Electron Microscope (SEM) was measured by Zeiss Gemini 300. The samples were dispersed in ethanol and spin-coated on a single crystal silicon substrate. Transmission Electron Microscope (TEM) were characterized by JEOL JEM 2100F at 200 kV accelerating voltage. Atomic Force Microscope (AFM, Bruker Dimension Edge) was operated in tapping mode at typical rate of 3 min per image. X-ray diffraction (XRD, X' Pert PRO MPD) patterns were recorded using Cu Kα radiation, with the angles from 5^o^ to 60^o^ and the rate of 2^o^ min^-1^. Raman spectra (Thermo Fischer DXR) had been taken from 100-2000 cm^-1^ at room temperature irradiated by 633 nm laser. X-ray photoelectron spectroscopy (XPS) analyses were conducted using a Thermo ESCALAB 250XI with a monochromatic Al Kα X-ray source (hv = 1487 eV). Photo-assisted Kelvin Probe Force Microscopy (KPFM) measurements were performed using an AFM system equipped with a SKPM module and a Xenon lamp, filtered at 420 nm. Electron Spin Resonance (ESR) spectroscopy, using a Bruker EMX Plus X-band at room temperature and 9.86 GHz, detected signals from unpaired electrons.

Density Functional Theory (DFT) calculations were executed using the Vienna ab initio simulation package (VASP). We employed the spin-polarized Generalized Gradient Approximation (GGA) in its Perdew-Burke-Ernzerhof (PBE) form for the exchange-correlation functional. The electron-core interactions were described using the Projector-Augmented Wave (PAW) method, and the plane wave basis set had a cutoff energy of 600 eV. For static, self-consistent calculations, a denser k-point mesh with a separation of 0.020 Å^−1^ was utilized. Structural relaxations were carried out using the conjugate gradient method, ensuring total energy convergence to 10^−6^ eV and atomic forces below 0.01 eV•Å^−1^. A 30 Å vacuum space along the z-direction was introduced to each system to prevent interactions between periodic structures.

The electronic structures were based on optimized lattice parameters and atomic positions. We used the Heyd-Scuseria-Ernzerhof (HSE06) hybrid functional to calculate the electronic structures of CeO_2_, pristine Nb_2_C, and Nb_2_C(OH)_2_. The Monkhorst−Pack k-point mesh was generated around the Γ point, with a spacing of 0.025 Å^−1^ for structural optimization. Additionally, the van der Waals interactions were accounted for using the DFT-D3 correction, enhancing the accuracy of interlayer interaction calculations. We also incorporated a dipole correction along the z-direction to eliminate errors in total energy, electrostatic potential, and atomic force arising from periodic boundary conditions.

**Animal Studies in the treatment of Achilles tendinopathy**

In the experiment investigating the effect of *Bmal1* enhancer Schottky heterojunctions on alleviating Achilles tendinitis symptoms in rats, we administered 50 μl of PBS, 50 μl of Nb_2_C (80 mg/ml), or 50 μl of Nb_2_C@CeO_2_ (80 mg/ml) via injection at the left Achilles tendon junction of each rat in the control, Nb_2_C, and Nb_2_C@CeO_2_ groups, respectively, at Zeitgeber time (ZT) 1 daily, following the completion of AT modeling. After 14 days, SD male rats were euthanized by cervical dislocation, and Achilles tendon tissue samples were collected from all groups at ZT1 for subsequent experiments.

In the experiment assessing the impact of different administration times on the efficacy of treating AT in rats, the modeled rats were divided into control, tendinopathy, Schottky (ZT13), and Schottky (ZT1) groups. AT was induced using established methods, and 50 μl of Nb_2_C@CeO_2_ (80 mg/ml) were injected at the left Achilles bone-tendon junction at the corresponding time points (ZT13/ZT1) daily. After 14 days, SD male rats were euthanized by cervical dislocation, and Achilles tendon tissue samples were collected from all groups at ZT1 for subsequent experiments.

**Cell viability assay**

The TDSCs were seeded in 96-well plates at a density of 5×103 cells/well and cultured at 37 ℃ with 5% CO2 for 24 hours, followed by the respective procedures. After incubation for 1, 3, and 5 days, each well was supplemented with 100 μL DMEM containing CCK-8 reagent (Bimake, China) at a concentration of 10% (v/v), followed by incubation at 37 ℃ for an additional hour. The optical density (OD) of each well was measured at a wavelength of 450 nm using a microplate reader (Thermo Fisher Scientific, USA).

**Live/Dead Staining**

After undergoing the corresponding processing, TDSCs were collected, subjected to three washes with PBS, and subsequently stained with calcein-AM/PI (Solarbio, China). Fluorescence images were observed using an inverted optical microscope (Olympus IMT-2, Tokyo, Japan).

**Elisa**

For detection of IL-6 and CRP in mouse serum, serum was isolated from whole blood and IL-6 and CRP was quantified by quantikine ELISA (R&D Systems, USA). Results are presented as mean ± SEM.

**Measurement of intracellular ROS levels**

TDSCs were seeded in six-well plates at a density of 2.0 ×10^5^ cells/well and subsequently exposed to TBHP with or without Nb2C or Schottky heterojunction. Following treatment, the cells were incubated with 10 mmol/L of 2′,7′-dichlorodihydrofluorescein diacetate (DCFH-DA) (Beyotime, Shanghai, China) for 30 minutes at 37 °C. ROS levels were assessed using fluorescence microscopy.

**Small Interfering RNA Transfection of TDSCs**

The TDSCs were transfected in 6-well plates and incubated with either control siRNA (si-ctrl; sense 5’-UUCUCCGAACGUGUCACGUTT-3’, antisense 5’-ACGUGACACGUUCGGAGAATT-3’) or Bmal1 siRNA (si-Bmal1; sense 5’-GGCACAUCGUGUUAUGAAUTT-3’, antisense 5’-AUUCAUAACACGAUGUGCCTT-3’) for a duration of 24 hours using Lipofectamine 3000 transfection reagent (Thermo Fisher, Logan, UT, USA) as per the manufacturer's instructions.

**Plasmid construction and transfection**

The pcDNA3.1(+)-Arntl-3xFLAG vectors used for overexpression assays were constructed and transfected into cells using a Lipofectamine 3000 transfection reagent (Thermo Fisher, Logan, UT, USA) according to the manufacturer’s instructions; empty vectors or plasmids carrying scrambled small-hairpin RNA were used as respective controls. The final efficiencies for overexpression were evaluated by PCR and western blotting.

**Luciferase assay**

The TDSCs were seeded in 96-well plates with an approximate confluence of 80% and subsequently transfected with luciferase reporter plasmids, pRL-TK-Renilla luciferase vectors (Promega), as well as the indicated expression vectors or compounds. The ratios between firefly and Renilla luciferase activities were quantified using a Dual-Luciferase Assay kit (Promega).

**Histopathologic analysis**

After a 14-day treatment period, all tendons from these groups were extracted and dehydrated using a gradient of ethanol. Subsequently, they were embedded in paraffin and sliced into 5 μm pieces. These sections underwent dewaxing with xylene and transitioned from the ethanol gradient to immersion in deionized water. Finally, the sections were subjected to HE, Masson, and Sirius staining following the manufacturer's instructions.

**Immunohistochemical staining**

After deparaffinization and rehydration, the tissue sections were permeabilized with 0.3% Triton X 100 (Beyotime Institute of Biotechnology) at room temperature for 10 minutes. Subsequently, they were blocked with 3% hydrogen peroxide. The sections were then treated with 0.4% pepsin (Sigma-Aldrich) in 1mM hydrochloric acid at a temperature of 37°C for one hour to retrieve the antigen. Following this, the sections were blocked again using 5% bovine serum albumin for a duration of thirty minutes at a temperature of 37 °C. Next, the sections were incubated overnight at a temperature of 4°C with the primary antibody and subsequently incubated with an HRP-conjugated secondary antibody for sixty minutes at a temperature of 37 °C. The subsequent step involved incubation with DAB (3,3-diaminobenzidine from Sigma-Aldrich), followed by counterstaining using hematoxylin. Positive expression in the cytoplasm and/or nucleus was observed as a brown stain, and images were captured using an inverted optical microscope (Olympus IMT-2, Tokyo, Japan).

**Immunofluorescence staining**

For TDSCs, the cells were washed with PBS three times, fixed in 4% paraformaldehyde for 15 minutes, and permeabilized with Triton X-100 for 15 minutes (0.1%). For tissue sections, the preliminary steps were identical to those of immunohistochemical staining. Following blocking with 3% bovine serum albumin at room temperature for 1 hour, the cells and sections were incubated overnight at 4°C with primary antibodies. Subsequently, they were washed with PBS and incubated at 37°C for 1 hour with FITC- or TRITC-conjugated secondary antibodies before being stained with DAPI for a duration of 5 minutes. Ten fields from each slide were randomly selected and observed under a fluorescence microscope (ZeissInc., Heidelberg, Germany).

**Real-time quantitative PCR**

The total RNA was extracted from TDSCs and tendon tissues using the RNA-Quick Purification Kit (Esunbio, Shanghai, China). Subsequently, cDNA synthesis was performed using the HiScript II Q RT SuperMix as a template for qPCR (Vazyme Biotech, Nanjing, China). For qPCR reactions, the ChamQTM SYBR Color qPCR Master Mix (Vazyme Biotech) was employed. The amplification process was carried out on a LightCycler 480-II instrument (Roche, Mannheim, Germany) utilizing the primers listed in Table S2.

**Western blot (WB) analysis**

The protein extraction from TDSCs and tendon tissues was performed using RIPA lysis buffer supplemented with 1 mM Phenylmethanesulfonyl fluoride (PMSF) and 1 mM protein phosphatase inhibitor. The protein concentration was determined using a BCA protein assay kit (Thermo Scientific). Subsequently, the proteins were separated by 10% SDS-PAGE and transferred onto Polyvinylidene Fluoride (PVDF) membranes (Millipore Co., Billerica, MA, USA). Then, the membranes were blocked with 5% (w/v) non-fat powdered milk for 1 h at room temperature, probed at 4 ℃ overnight with primary antibody against COL1 (1:1000, Proteintech), COL3 (1:1000, Proteintech), MMP3 (1:1000, Proteintech), MMP13 (1:1000, Proteintech), NOX1 (1:1000, Proteintech), NOX4 (1:1000, Proteintech), SOD1 (1:1000, Proteintech), β-ACTIN (1:2000, Cell Signaling Technology), GAPDH (1:2000, Abcam), NRF2 (1:1000, Abcam), KEAP1 (1:2000, ABclonal), HO-1 (1:500, Proteintech), HISTONE H3 (1:1000, Abcam) and BMAL1 (1:1000, Proteintech). After being washed three times with TBS containing 0.05% Tween 20 (TBST), the membranes were incubated with horseradish peroxidase-conjugated secondary antibodies for a duration of 1 hour. The protein level was detected using the ChemiDocXRS + Imaging System (Tanon, Shanghai, China).

**Statistical analysis**

Statistical analyses were performed using GraphPad Prim 8.0 software. All data are presented as mean values ± SD, and a P value <0.05 was considered statistically significant. Unpaired t-tests were used to analyze differences between two groups. One-way ANOVA with Tukey's multiple comparison test was used for comparisons among more than two groups. Pearson's rank correlation test was used to assess correlations. Statistical parameters are described in the figure legends. Unless otherwise specified, all experiments were conducted using three independent samples.


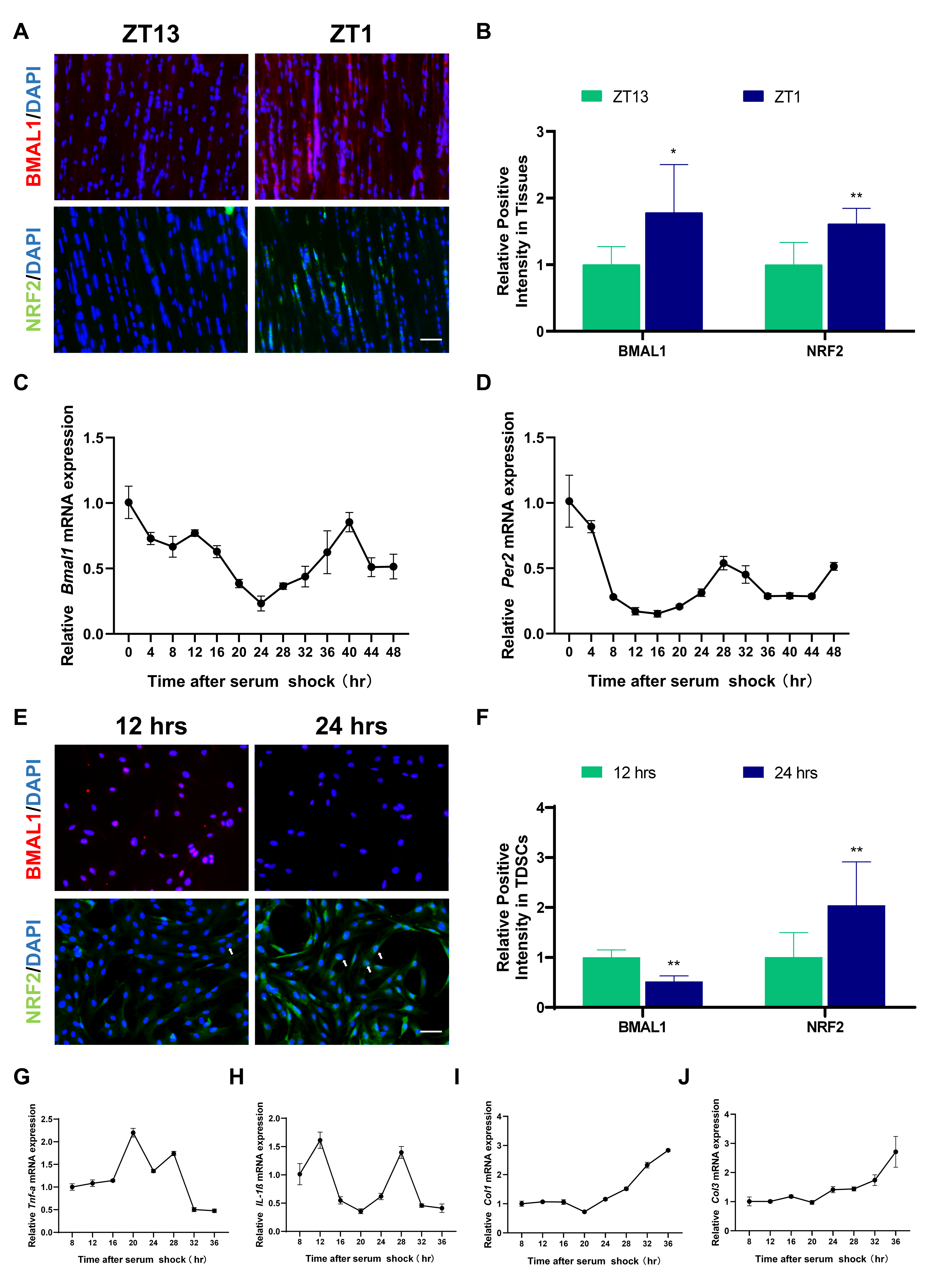


Fig. S1. The Achilles tendon functions as a peripheral oscillator. (A and B) The immunofluorescence staining analysis for BMAL1 and NRF2 in ZT13 and ZT1 groups. (C and D) The mRNA expression of *Bmal1* and *Nrf2* at 4-hour intervals over a 48-hour period in TDSCs. (E and F) The immunofluorescence staining analysis for BMAL1 and NRF2 in TDSCs at 12 or 24 hours after serum shock. (G to J) The mRNA expression of *TNF-α, IL-1β, Col1,* and *Col3* at 4-hour intervals over a 28-hour period in TDSCs. Data are presented as the mean ± SD. Scale bar, 100 μm.


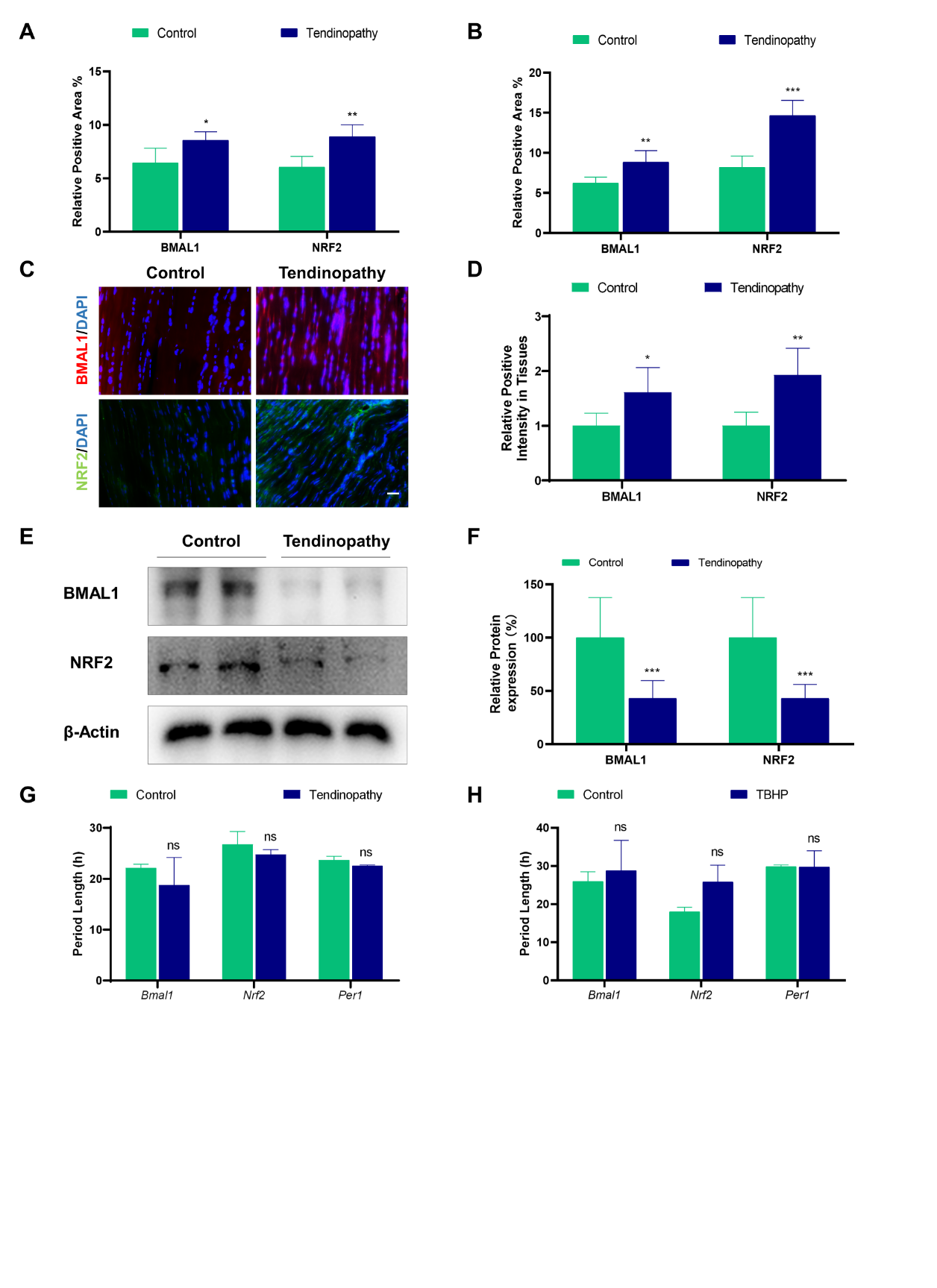


Fig. S2. Unraveling a disrupted circadian clock in Achilles tendinopathy. (A) Quantitative analysis of immunochemistry analysis for BMAL1 and NRF2 in human Achilles tendon tissues with or without tendinopathy. (B) Quantitative analysis of immunochemistry analysis for BMAL1 and NRF2 in rat Achilles tendon tissues with or without tendinopathy. (C and D) The immunofluorescence staining analysis for BMAL1 and NRF2 in rat Achilles tendon tissues with or without tendinopathy. (E) Western blot analysis of BMAL1 and NRF2 in rat Achilles tendon tissues with or without end-stage tendinopathy. (F) Quantification of the Western blot data from C. (G) Period length of circadian rhythm expression of *Bmal1*, *Nrf2* and *Per1* in rat Achilles tendon tissues with or without tendinopathy. (H) Period length of circadian rhythm expression of *Bmal1*, *Nrf2,* and *Per1* in TDSCs treated with or without TBHP. Data are presented as the mean ± SD. *p < 0.05, **p < 0.01, ***p < 0.001; ns, not significant. Scale bar, 100 μm.


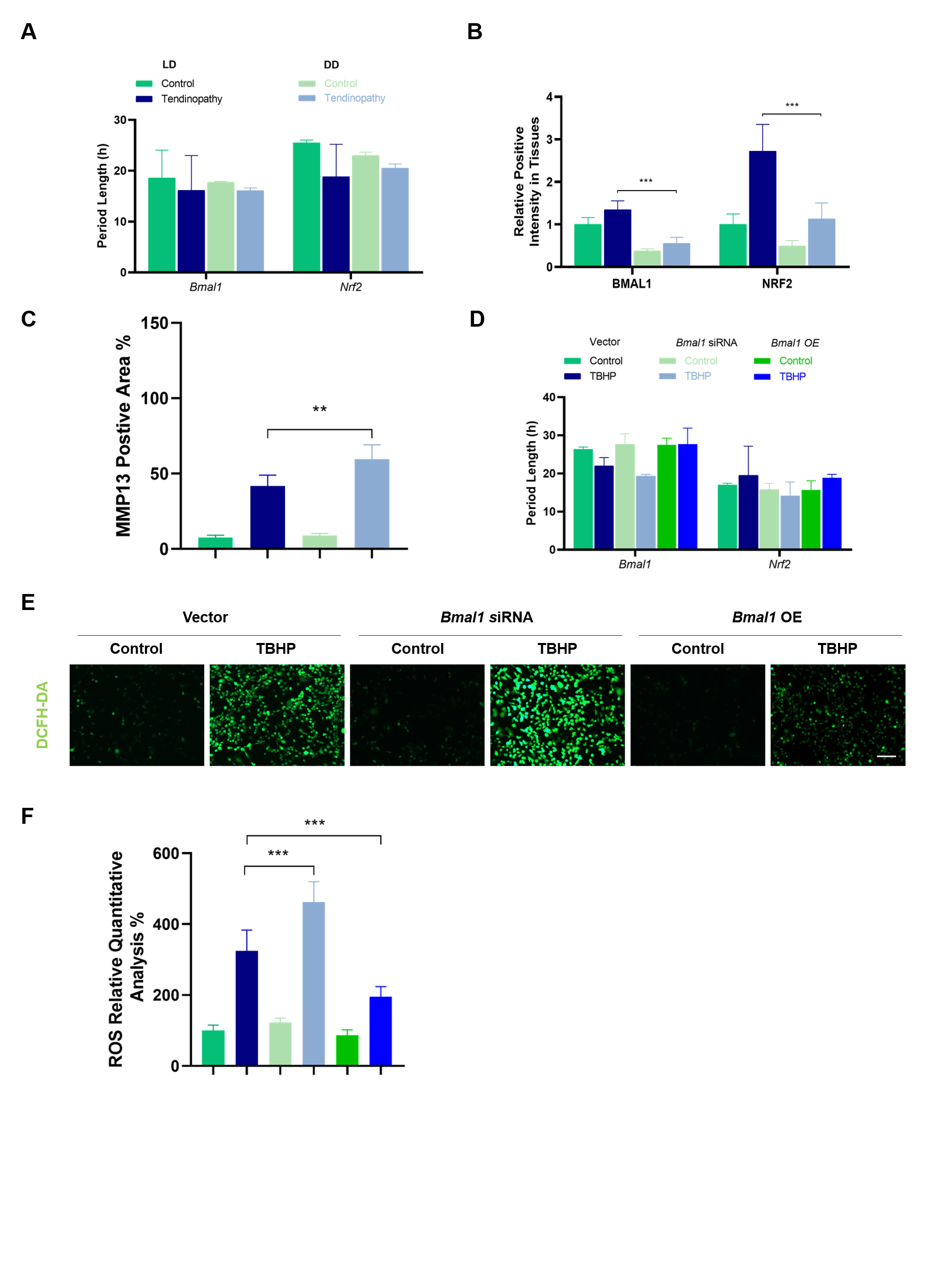


**Fig. S3**. *Bmal1* is a potential therapeutic target for Achilles tendinopathy. (A) Period length of circadian rhythm expression of *Bmal1* and *Nrf2* in rat Achilles tendon tissues with or without tendinopathy under LD or DD conditions. (B) Relative quantitative analysis data of immunofluorescence of BMAL1 and NRF2 in Figure 3G. (C) Relative quantitative analysis data of immunohistochemistry staining of MMP13 in Figure 3H. (D) Period length of circadian rhythm expression of *Bmal1* and *Nrf2* in TDSCs treated with *Bmal1* siRNAs or *Bmal1* overexpression plasmid with or without TBHP. (E and F) The ROS staining analysis in the six groups. Data are presented as the mean ± SD. *p < 0.05, **p < 0.01, ***p < 0.001; ns, not significant. Scale bar, 100 μm.


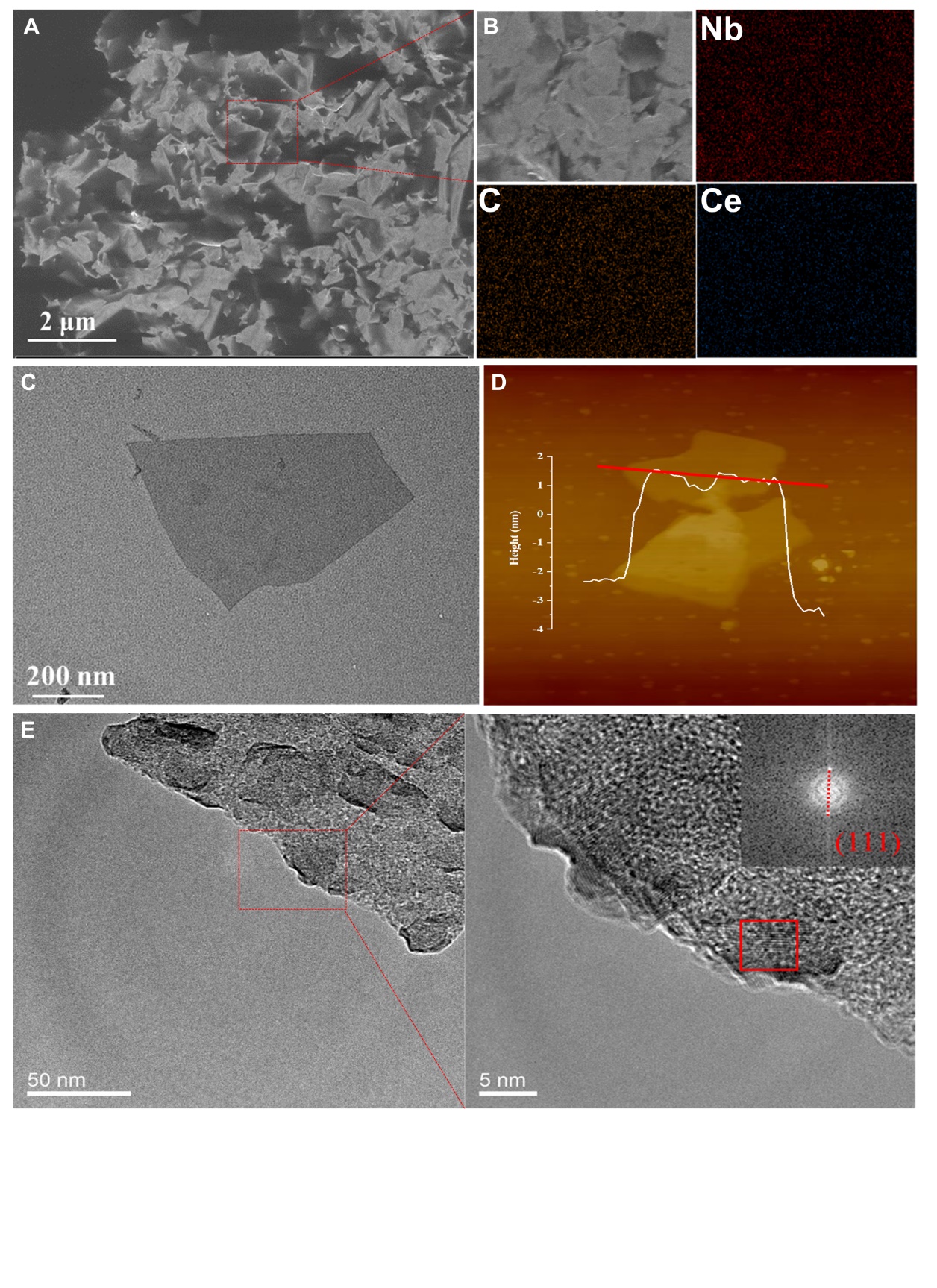


Fig. S4. External morphology of as-fabricated Schottky heterojunction. (A) SEM image of Nb_2_C@CeO_2_ (Schottky heterojunction). (B) The in-situ sectional Energy Dispersive Spectrometer mapping. (C) TEM pattern of ultrathin Nb_2_C treated by chemical exfoliation. (D) AFM image and height profile corresponds to the red outline of Nb_2_C@CeO_2_ (Schottky heterojunction). (E) TEM image of Nb_2_C@CeO_2_ (Schottky heterojunction). And the partial lattice fringes were analyzed by live FFT. Data are presented as the mean ± SD. Scale bar, 10 nm.


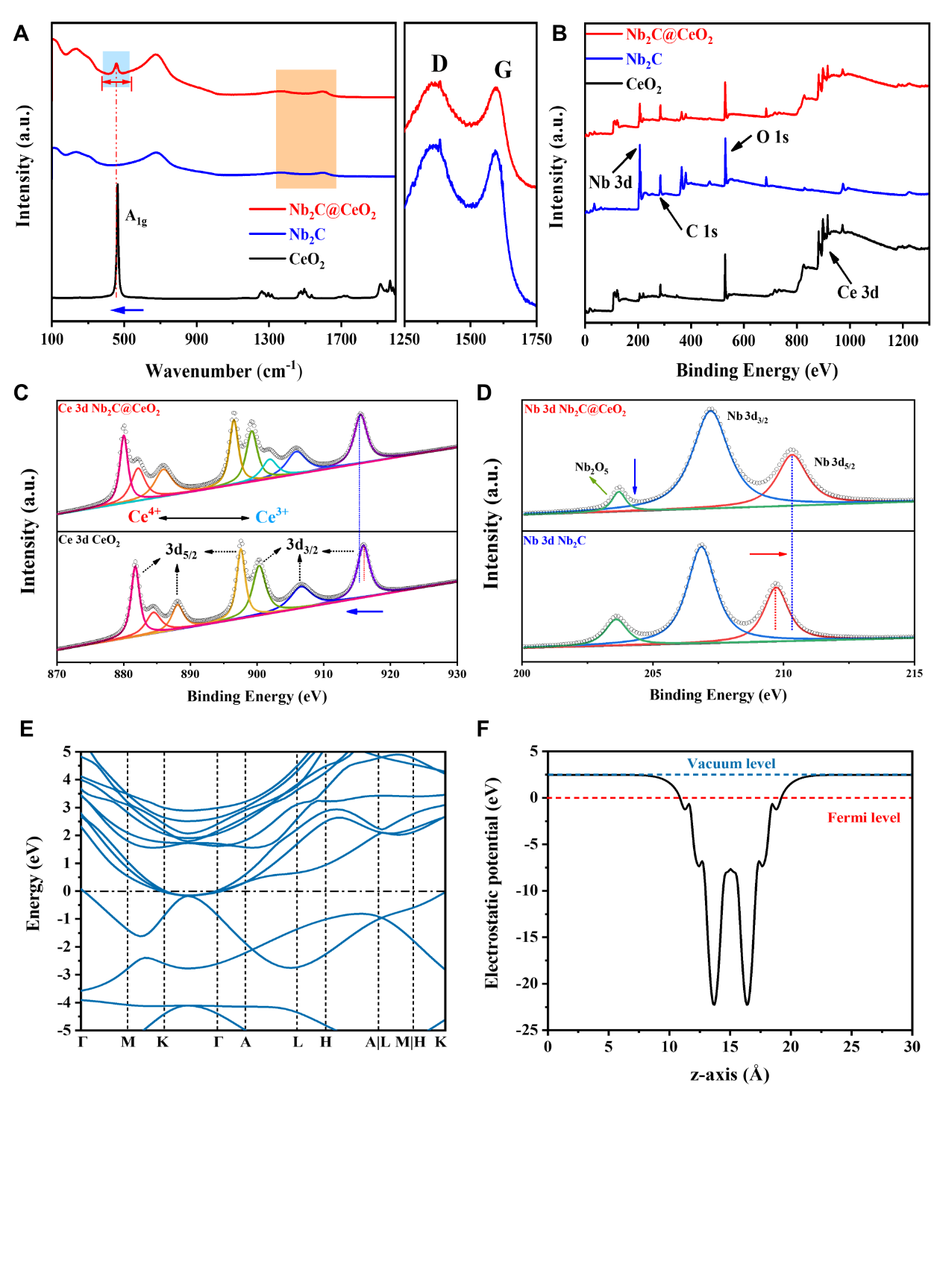


Fig. S5. Internal bonding and electrical transfer stemmed on Schottky heterojunction. (A) Raman spectra of CeO_2_, Nb_2_C, and Nb_2_C@CeO_2_ (Schottky heterojunction). (B) XPS survey of CeO_2_, Nb_2_C, and Nb_2_C@CeO_2_ (Schottky heterojunction). The XPS survey indicates the overall situation, in which both Nb_2_C and Nb_2_C@CeO_2_ (Schottky heterojunction) reveal the signals of C 1s, O 1s and Nb 3d. Aggregated XPS spectra (C and D) Ce 3d and Nb 3d with fitted curves. (E) Band structure of Nb_2_C(OH) calculated by *HSE06*. (F) The planar average potential along z-axis and work function of Nb_2_C(OH). Data are presented as the mean ± SD.


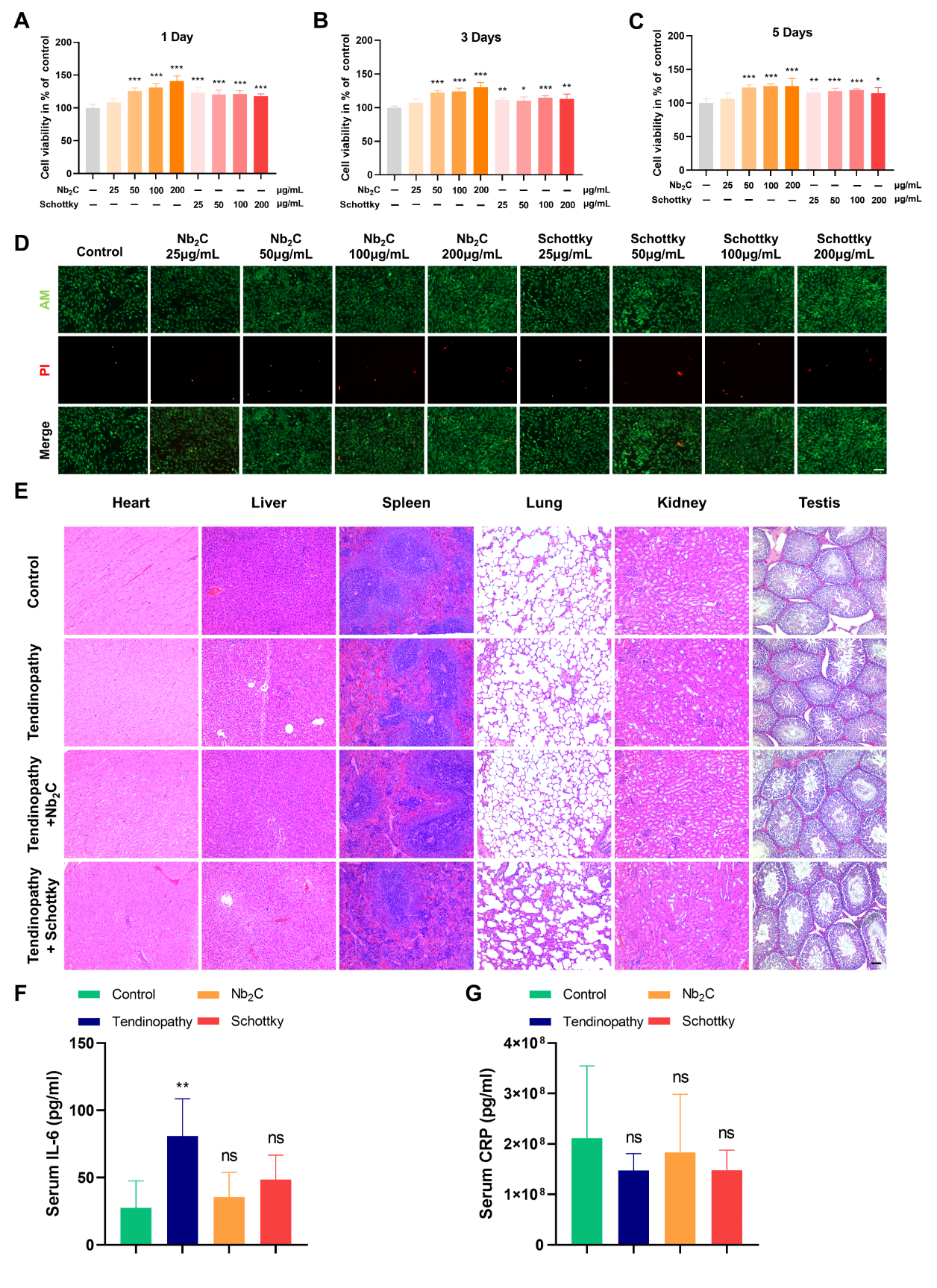


Fig. S6. Nb_2_C and Schottky heterojunction have great biocompatibility. (A to C) The CCK-8 analysis in TDSCs treated with Nb_2_C or Schottky heterojunction. (D) Representative images of the Live/Dead Staining in TDSCs after treatment with Nb_2_C or Schottky heterojunction respectively. (E) Representative images of the HE staining in rat heart, liver, spleen, lung, kidney and testis tissues treat with or without Nb_2_C or Schottky heterojunction. (F and G) Elisa assay of IL-6 and CRP in the serum of rat treat with or without Nb_2_C or Schottky heterojunction. Data are presented as the mean ± SD. *p < 0.05, **p < 0.01, ***p < 0.001; ns, not significant. Scale bar, 100 μm.


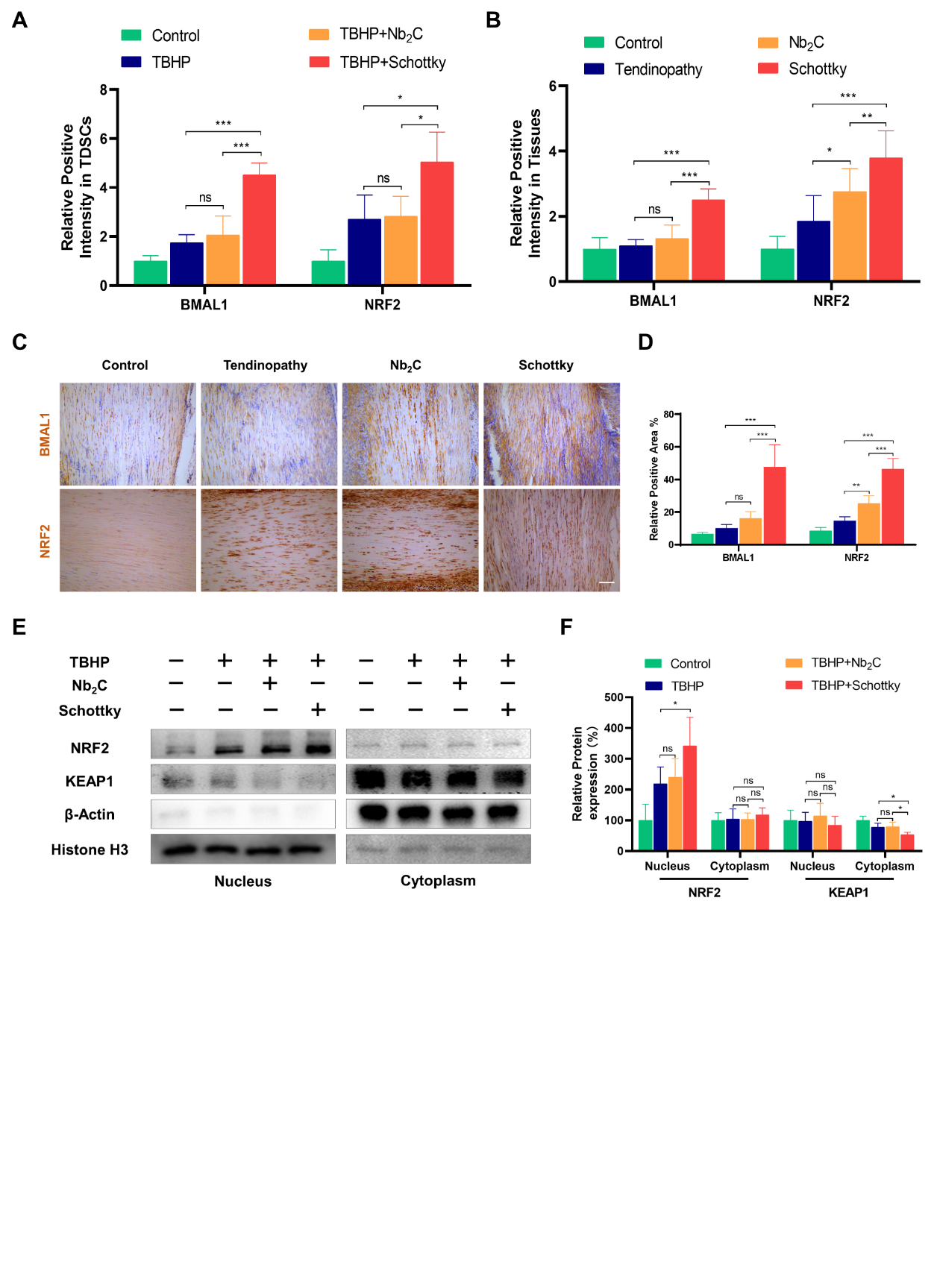


Fig. S7. *Bmal1* enhancer Schottky heterojunction increase *Bmal1* expression and activates *Nrf2*-driven antioxidant pathway in TDSCs and tendon tissues. (A) Relative quantitative analysis data of Immunofluorescence staining for BMAL1 and NRF2 in the in Figure 5D. (B) Relative quantitative analysis data of immunofluorescence staining for BMAL1 and NRF2 in in Figure 5E. (C and D) Immunohistochemistry staining for BMAL1 and NRF2 in rat Achilles tendon tissues treated with or without Nb_2_C or Schottky heterojunction. (E and F) Western blot analysis for nuclear and cytoplasmic expression of NRF2 and KEAP1 in TDSCs in the four groups.  Data are presented as the mean ± SD. *p < 0.05, **p < 0.01, ***p < 0.001; ns, not significant. Scale bar, 100 μm.


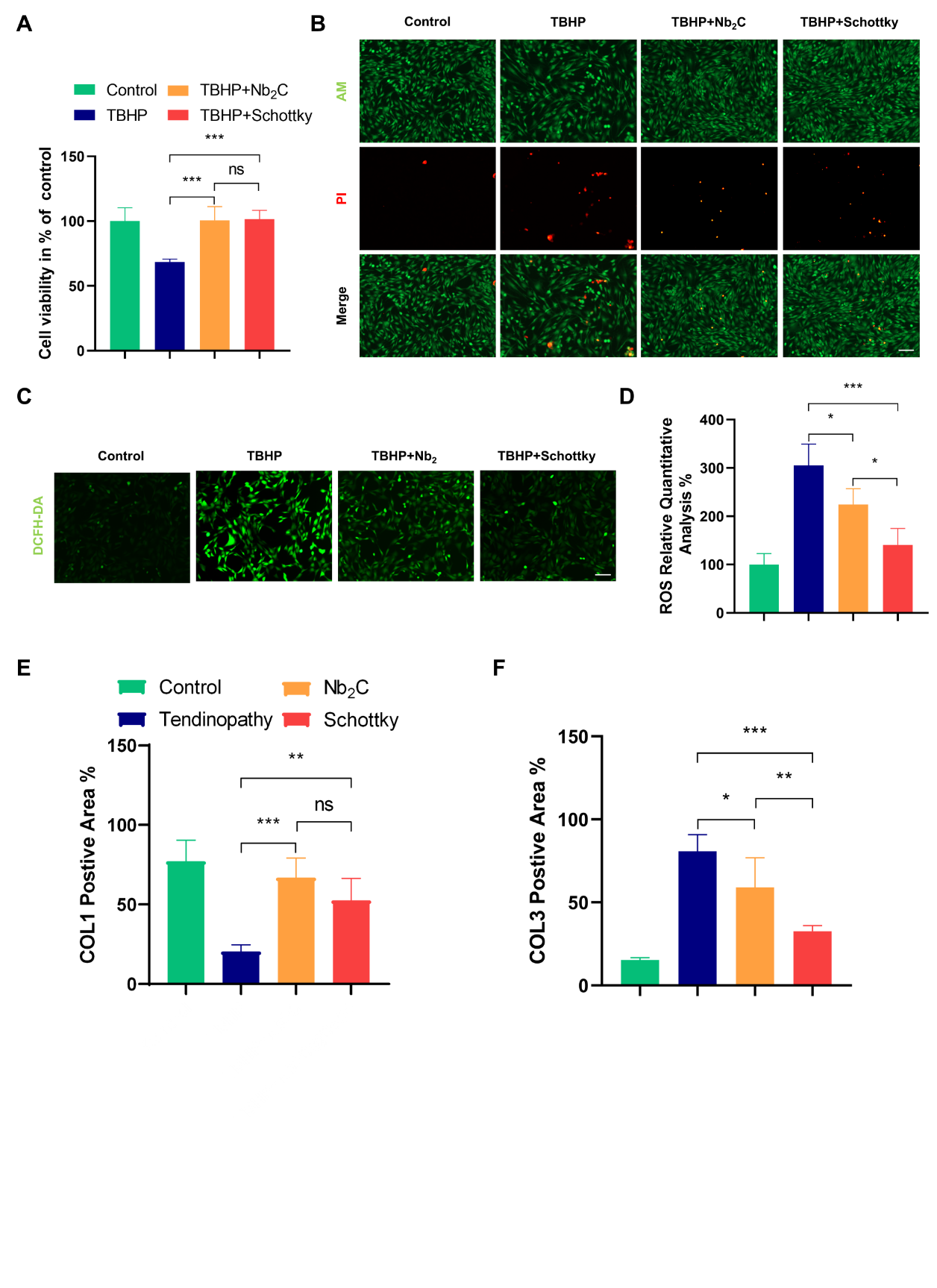


Fig. S8. *Bmal1* enhancer Schottky heterojunction ameliorate tendon deterioration in Achilles tendinopathy in vitro and in vivo. (A) The viability of TDSCs after TBHP treatment and treatment with or without Nb_2_C or Schottky heterojunction for 6h.(B) Representative images of the Live/Dead Staining in TDSCs after TBHP treatment and treatment with or without Nb_2_C or Schottky heterojunction respectively.(C) In each group, fluorescence images of the DCFH-DA probe for hydrogen peroxide in TDSCs. (D) ROS relative quantitative analysis data from Figure S8C.(E-F) Relative quantitative analysis data of immunohistochemistry staining of COL1 and COL3 in Figure 5L. Data are presented as the mean ± SD. *p < 0.05, **p < 0.01, ***p < 0.001; ns, not significant. Scale bar, 100 μm.


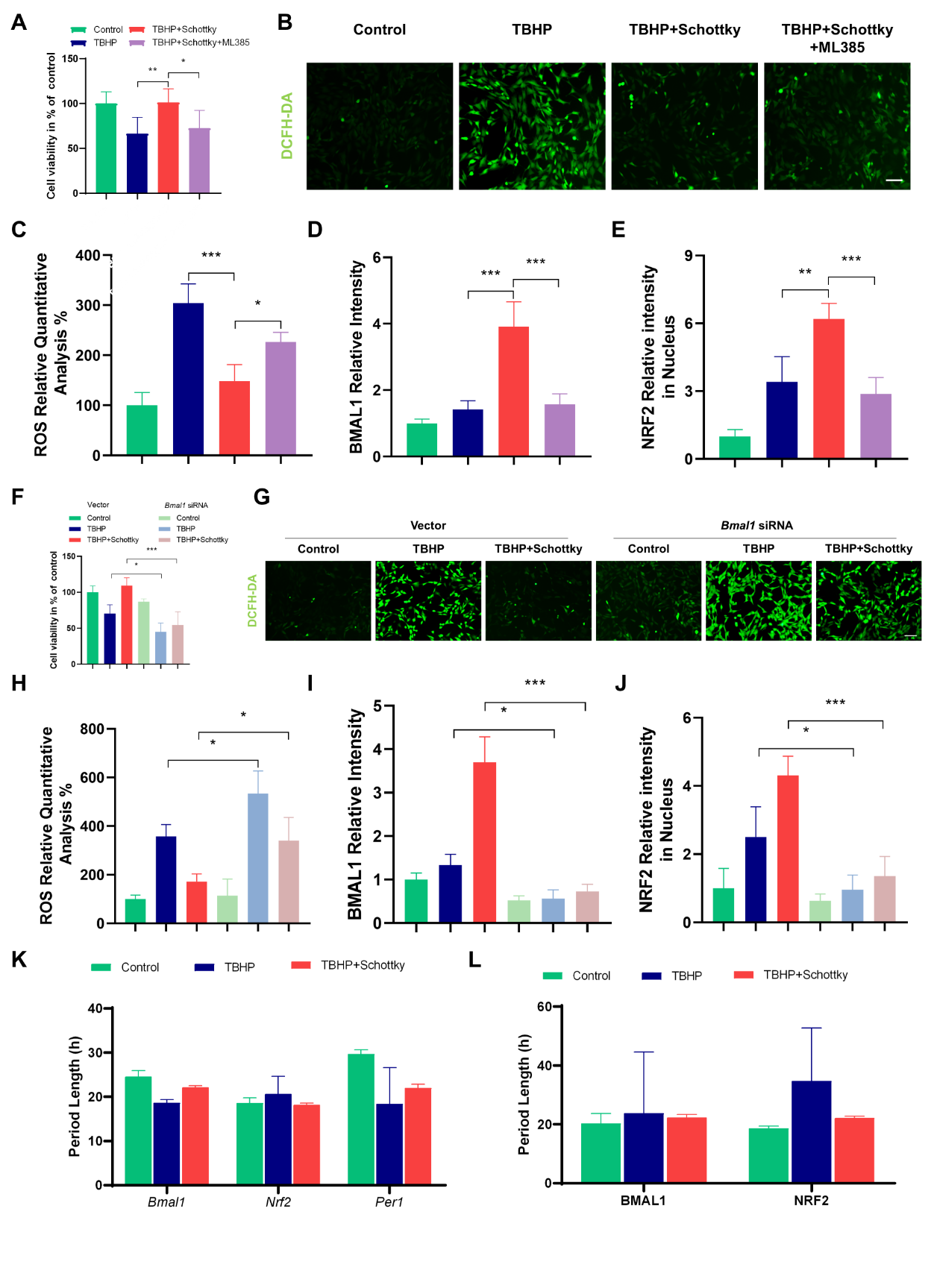


Fig. S9. Schottky heterojunction exerts therapeutic effects by regulating the circadian clock in Achilles tendons and subsequently modulating the *Bmal1*-regulated *Nrf2*-mediated antioxidant stress mechanism. (A) The viability of TDSCs after TBHP treatment, treatment with or without Schottky heterojunction treatment, and treatment with or without ML385. (B) Fluorescence images of the DCFH-DA probe for hydrogen peroxide in TDSCs in each group. (C) ROS relative quantitative analysis data from D. (D) Quantification of the immunofluorescence staining data for BMAL1 in TDSCs after TBHP treatment, treatment with or without Schottky heterojunction treatment, and treatment with or without ML385. (E) Quantification of the immunofluorescence staining data for NRF2 in TDSCs after TBHP treatment, treatment with or without Schottky heterojunction treatment, and treatment with or without ML385. (F) The viability of TDSCs transfected with *Bmal1*-siRNA after TBHP treatment and treatment with or without Schottky heterojunction. (G) Fluorescence images of the DCFH-DA probe for hydrogen peroxide in TDSCs in each group. (H) ROS relative quantitative analysis data from D. (I) Quantification of the immunofluorescence staining data for BMAL1 in TDSCs transfected with *Bmal1*-siRNA after TBHP treatment and treatment with or without Schottky heterojunction. (J) Quantification of the immunofluorescence staining data for NRF2 in TDSCs transfected with *Bmal1*-siRNA after TBHP treatment and treatment with or without Schottky heterojunction. (K) Period length of mRNA circadian rhythm expression of *Bmal1*, *Nrf2* and *Per1* in TDSCs treat with or without TBHP after treatment with or without Schottky heterojunction. (L) Period length of protein circadian rhythm expression of BMAL1 and NRF2 in TDSCs treat with or without TBHP after treatment with or without Schottky heterojunction. Data are presented as the mean ± SD. *p < 0.05, **p < 0.01, ***p < 0.001; ns, not significant. Scale bar, 100 μm.

Table S1. Clinical information regarding the patients from whom tendon samples were obtained.

| **Group** | **Diagnosis** | **Gender** | **Age**  **(years)** | **Sampling times** |
| --- | --- | --- | --- | --- |
| Control | Aortic dissection | Male | 45 | 14:00-14:40 |
|  | Artery and nerve injury of right lower extremity | Male | 47 | 15:15-16:15 |
|  | Multiple trauma | Male | 44 | 12:45-13:45 |
| Tendinopathy | Right rotator cuff injury | Male | 45 | 17:30-18:30 |
|  | Rheumatoid arthritis | Female | 50 | 9:00-10:35 |
|  | Adhesive capsulitis of the shoulder | Female | 44 | 12:00-12:30 |

Table S2. Primers used for qPCR.

| **Oligonucleotide** | **Sequence from 5ʹ to 3ʹ** | **Oligonucleotide** | **Sequence from 5ʹ to 3ʹ** |
| --- | --- | --- | --- |
| R-β-actin-F | CAGGGTGTGATGGTGGGTATGG | R-IL-1β-F | CAGCTTTCGACAGTGAGGAGA |
| R-β-actin-R | AGTTGGTGACAATGCCGTGTTC | R-IL-1β-R | CTCCACGGGCAAGACATAGG |
| R-Bmal1-F | TATCACACTACGAAGTCGATGG | R-Col1a1-F | GAAGAGGAGATAGAGCGAAACA |
| R-Bmal1-R | CGGCAATCATTCGACCTATTTT | R-Col1a1-R | CAATCTTCTTCAGGACCTGACC |
| R-Nrf2-F | TAGATCTTGGGGTAAGTCGAGA | R-Col3a1-F | GATCGTGGTGAAAATGGTTCTC |
| R-Nrf2-R | CTCTTGTCTCTCCTTTTCGAGT | R-Col3a1-R | GTTCACCAGTTTCACCTTTGTC |
| R-Per1-F | AACATTCCTAACACAACCAAGC | H-β-ACTIN-F | AATCGTGCGTGACATTAAGGAG |
| R-Per1-R | CTGCTGACGACGTATCTTTCTT | H-β-ACTIN-R | ACTGTGTTGGCGTACAGGTCTT |
| R-Per2-F | CTCTGACACATCCCAGTCTAG | H-MMP3-F | AGTCTTCCAATCCTACTGTTGCT |
| R-Per2-R | GTCCCTGGTGTGGATACTATTC | H-MMP3-R | TCCCCGTCACCTCCAATCC |
| R-Mmp3-F | GCTGTCTTTGAAGCATTTGGGTT | H-TNF-α-F | CCTCTCTCTAATCAGCCCTCTG |
| R-Mmp3-R | CCCTCCATGAAAAGACTCAGAGG | H-TNF-α-R | GAGGACCTGGGAGTAGATGAG |
| R-Tnf-α-F | ATGGGCTCCCTCTCATCAGT | H-IL-6-F | ACTCACCTCTTCAGAACGAATTG |
| R-Tnf-α-R | GCTTGGTGGTTTGCTACGAC | H-IL-6-R | CCATCTTTGGAAGGTTCAGGTTG |
| R-IL-6-F | AGAGACTTCCAGCCAGTTGC |  |  |
| R-IL-6-R | TGCCATTGCACAACTCTTTTC |  |  |
